# Nanovesicle-Mediated Delivery Systems for CRISPR/Cas Genome Editing

**DOI:** 10.3390/pharmaceutics12121233

**Published:** 2020-12-18

**Authors:** Dongyoon Kim, Quoc-Viet Le, Yina Wu, Jinwon Park, Yu-Kyoung Oh

**Affiliations:** College of Pharmacy and Research Institute of Pharmaceutical Sciences, Seoul National University, Seoul 08826, Korea; mmamic@snu.ac.kr (D.K.); lqviet.pharm@gmail.com (Q.-V.L.); yena-oh@snu.ac.kr (Y.W.); pjw6846@snu.ac.kr (J.P.)

**Keywords:** genome editing, CRISPR/Cas9, sgRNA, nonviral vectors, nanovesicles

## Abstract

Genome-editing technology has emerged as a potential tool for treating incurable diseases for which few therapeutic modalities are available. In particular, discovery of the clustered regularly interspaced short palindromic repeats (CRISPR)/Cas system together with the design of single-guide RNAs (sgRNAs) has sparked medical applications of genome editing. Despite the great promise of the CRISPR/Cas system, its clinical application is limited, in large part, by the lack of adequate delivery technology. To overcome this limitation, researchers have investigated various systems, including viral and nonviral vectors, for delivery of CRISPR/Cas and sgRNA into cells. Among nonviral delivery systems that have been studied are nanovesicles based on lipids, polymers, peptides, and extracellular vesicles. These nanovesicles have been designed to increase the delivery of CRISPR/Cas and sgRNA through endosome escape or using various stimuli such as light, pH, and environmental features. This review covers the latest research trends in nonviral, nanovesicle-based delivery systems that are being applied to genome-editing technology and suggests directions for future progress.

## 1. Introduction

Gene therapy, which holds the promise of fundamentally curing diseases through the introduction of exogenous genetic material, has recently emerged as a new alternative to conventional disease treatments [1,2,3]. Building on the central dogma that genetic information flows from DNA to RNA to protein, developed by Francis Crick in 1958, researchers have attempted to induce more sustained and improved therapeutic effects by replacing defective proteins through introduction of DNA or mRNA that can express normal proteins [4,5,6]. Moreover, as molecular biology and genetics have advanced, RNA interference mediated by small inhibitory RNA (siRNA) or microRNA (miRNA) has also become a powerful tool for gene therapy [7,8,9]. Thus, gene therapy has shown great therapeutic potential in various diseases that were once considered undruggable [10,11].

The concept of genome surgery, in which harmful genes are replaced with normal genes, is a more advanced form of gene therapy in that it leads to changes in the genome. During DNA repair, DNA cleaved out at a desired gene locus is restored by nonhomologous end joining or homology-directed repair [12]. These pathways enable insertion of specific new functional genes or suppression of existing genes [13,14]. Until the early 2010s, such genome engineering was mainly carried out using a specialized enzyme called zinc-finger nuclease (ZFN) and transcription activator-like effector nucleases (TALENs) [15,16]. By conjugating a specific DNA-binding protein domain to a DNA domain that can be cleaved by the restriction endonuclease, *Fok*I, both ZFN and TALEN can perform specific site-guided DNA cleavage.

Clinical trials are ongoing using these programmable nucleases. ZFN-based genome editing is in clinical trial for treatment of human immunodeficiency virus infection (NCT00842634). In this study, ZFN was used to delete CC chemokine receptor 5, which is a coreceptor for human immunodeficiency virus entry on CD4 T cells. Another ZFN-mediated genome editing technology is also in clinical trial for treatment of hemophilia B (NCT02695160). In this trial, ZFN was used to insert Factor IX gene into the genome of hepatocytes. TALEN-mediated genome editing is also in clinical trial for treatment of refractory B-cell acute lymphoplastic leukemia (NCT04150497). TALEN was used to insert anti-CD22 chimeric antigen receptor gene into the genome of T cells. Although both ZFN and TALEN have shown promising therapeutic effects against various diseases, the protein nuclease enzyme engineering is time consuming, and difficult. Construction of a target-recognizing protein array and linkage of the array to the nucleases requires multistep processes and experiences. Moreover, choosing a suitable target DNA site is sometimes problematic [17]. This technical complexity has limited the applicability of the ZFN/TALEN platform [18,19,20].

The discovery of clustered regularly interspaced short palindromic repeats (CRISPR) and CRISPR-associated protein (Cas) has revolutionized the concept of genome editing. CRISPR/Cas was originally discovered in bacteria, where it serves an immune system-like function, acting as a defense mechanism against bacteriophages [21]. After degrading the genomes of invaded bacteriophages, bacteria acquire the resulting DNA fragments and insert them into their genomes as spacers in the CRISPR locus. Subsequent transcription of the CRISPR region and RNA processing produces a spacer sequence harboring small RNAs called CRISPR RNAs (crRNAs) and trans-activating crRNAs (tracrRNAs), which together constitute the single-guide RNA (sgRNA). This sgRNA, in turn, forms a complex with Cas protein, which possesses nuclease activity, after which the sgRNA mediates recognition of the specific DNA spacer sequence. By providing a means for bacteria to cut the bacteriophage genome at sites corresponding to the sgRNA sequence, this process allows bacteria to acquire adaptive immunity against bacteriophages [22].

In principle, by complexing different unique sgRNA sequences with Cas9 protein—one of the most popular Cas family types used for gene editing—the CRISPR/Cas9 system provides a much simpler and easier method for constructing a programmable nuclease than previous genome-editing tools [23]. Moreover, the CRISPR/Cas9 system is more flexible with respect to choosing a target DNA sequence [24]. Given this versatility, CRISPR/Cas9-mediated precise genome-editing technology has moved to the forefront in various fields, including disease treatment, diagnosis, drug discovery, and animal model development, among others [25]. Such remarkable potential value led CRISPR/Cas9 gene-editing technology to be awarded the 2020 Nobel Prize in chemistry less than 8 years after its creation.

Despite advances in the development of CRISPR/Cas9, widespread clinical application is not on the immediate horizon. The biggest factor hampering the virtually unlimited potential of genome engineering is the lack of effective delivery systems. Conventional viral delivery systems based on adenoviruses or retroviruses possess high transfection efficiency, but their inherent toxicity, including induction of immune responses and insertion of viral genomes into host chromosomes, has limited their use [26,27]. Adeno-associated viruses are known to be safer, but their small packaging capacity is a constraint on the delivery of CRISPR/Cas9 components [28]. On the other hand, nonviral delivery systems are safer and are not restricted by the size of genome-editing components, but their delivery efficiency is relatively low [29].

With recent advances in nanotechnology, a variety of nonviral delivery systems have been developed for genome editing. Systems for CRISPR/Cas9 delivery include synthetic nanoparticles based on liposomes, polymers, or polypeptides, as well as natural extracellular vesicles. In addition, various strategies have been developed for enhancing delivery efficacy, such as facilitating endosomal escape, active targeting, and stimulus-responsive cargo release. This review summarizes recent trends in nanomaterials and delivery strategies used in genome engineering, and presents future perspectives on genome-engineering technology as a next-generation medicine.

## 2. Nonviral Delivery Systems for Genome Editing

**Figure 1:**
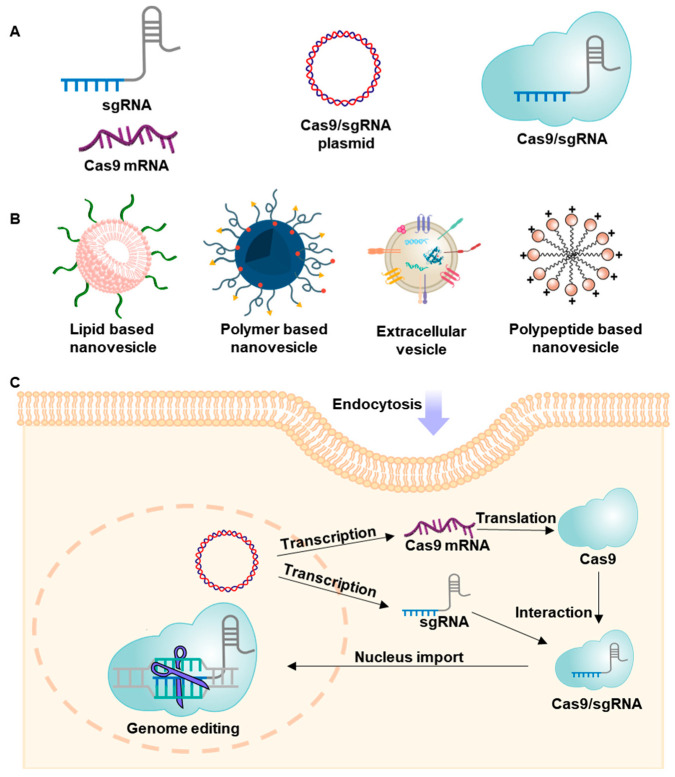
Illustration of nanovesicle types for CRISPR/Cas9 and sgRNA delivery. (**A**) Different forms of the CRISPR/Cas9 system. (**B**) Types of delivery systems for CRISPR/Cas9 and sgRNA. (**C**) Genome-editing mechanisms of nanovesicle-delivered Cas9/sgRNA plasmid DNA.

The CRISPR/Cas9 system can be delivered to cells in the form of DNA, RNA, or protein, depending on the purpose, and various delivery systems based on these forms have been designed. Delivery systems can be broadly categorized into lipids, polymers, peptide/protein nanovesicles, and extracellular vesicles (Figure 1, Table 1).

### 2.1. Lipid-Based Vesicles for Genome Editing

#### 2.1.1. Cationic Lipid Nanoparticles

**Figure 2:**
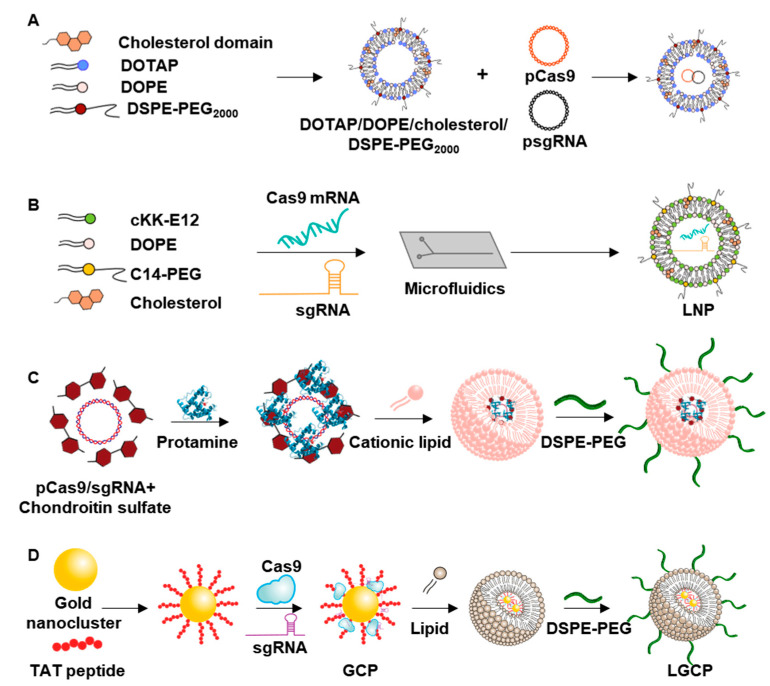
Lipid-based systems for CRISPR/Cas9 and sgRNA delivery. (**A**) Cholesterol-rich liposome for delivery of plasmid DNA encoding Cas9 (pCas9) and plasmid DNA encoding sgRNA (psgRNA). Adapted from [53], Dovepress, 2019. (**B**) cKK-E12–containing cationic liposome for Cas9 mRNA and sgRNA delivery. Adapted from [32], Nature Publishing Group, 2017. (**C**) Cationic liposome for pCas9/sgRNA delivery. Adapted from [33], Springer Nature, 2017. (**D**) Gold nanocluster/lipid core-shell nanoparticle for Cas9/sgRNA delivery. Adapted from [34] WILEY-VCH, 2017.

Lipids are one of the most extensively used materials for nonviral gene-delivery systems [49,50,51]. Most lipids studied for gene delivery are amphiphilic compounds bearing hydrophobic tails and hydrophilic head groups. These amphipathic lipids self-assemble in a physiological aqueous environment, forming vesicles with a bilayer structure. Cationic liposomes in particular have been studied for gene delivery owing to their potential charge-charge complexation with anionic nucleic acids or CRISPR/Cas9 ribonucleoprotein complexes. These lipid-based vesicles have been reported to reduce susceptibility of nucleic acids to degradation by nucleases [52]. Representative examples of lipid-based nanovesicles are illustrated in Figure 2.

Among cationic lipids, the quaternary amine group-containing 1,2-dioleoyl-3-trimethylammoniumpropane (DOTAP) has been widely used as a component of cationic liposomes [54]. Polyethylene glycol (PEG)-modified DOTAP-based cationic liposomes have been studied for delivery of sgRNA specific for enhanced green fluorescent protein (EGFP) and plasmid DNA encoding Cas9 (pCas9) (Figure 2A) [53]. These liposomes were prepared from DOTAP, 1,2-dioleoyl-sn-glycero-3-phosphoethanolamine (DOPE), cholesterol and 1,2-distearoyl-sn-glycero-3-phosphoethanolamine-N-[amino(polyethyleneglycol)-2000] (DSPE-PEG-2000) or PEG-linked cholesterol (Chol-PEG). The use of DOPE was found to increase the transfection efficiency of cationic liposomes. Delivery of these liposomes into EGFP-expressing HEK293 cells resulted in gene-editing activity, as evidenced by decreased expression of the sgRNA target gene, EGFP.

Another study utilized DOTAP-based cationic liposome for delivery of pCas9 and viral protein-specific sgRNA to treat cervical cancer [30]. In this study, liposomes composed of DOTAP, DOPE, cholesterol, and DSPE-PEG-2000 were complexed with human papillomavirus 16 E6/E7 (HPV 16 E6/E7)-specific sgRNA and pCas9. Treatment of SiHa human cervical cancer cells with lipoplexes was shown to suppress expression of the target viral proteins, E6 and E7. In this study, six repeated intravenous injections of lipoplexes at 3-day intervals caused no histologically evident toxicity or changes in inflammatory cytokine levels.

Cationic liposomes have also been studied for treatment of the genetic disease, mucopolysaccharidosis type I [31], a lysosomal storage disease caused by a deficiency of the enzyme, α-L-iduronidase (IDUA) [55]. In this study, three components—pCas9, sgRNA, and cDNA encoding IDUA—were complexed with cationic liposomes and intravenously delivered into IDUA-knockout mice. This treatment was found to improve IDUA activity in vivo.

In addition to DOTAP, a palm stearin-based cationic lipid nanocarrier has been used to form lipid nanoparticles [56]. These nanoparticles, containing the fatty acids myristate, palmitate, stearate, oleate, and linoleate, were complexed with plasmid DNA encoding Cas9 and sgRNA targeting the reporter gene, *EGFP.* Treatment of EGFP-expressing HEK 293 cells with palmstearin-based lipoplexes reduced the expression of EGFP to a greater extent than a palmitic acid-based cationic lipid nanocarrier.

Lysine-based cationic peptide conjugation has been studied for its potential to confer a cationic feature onto lipid nanoparticles for the delivery of genome-editing components (Figure 2B) [32]. In this study, the cationic peptide, cKK-E12, was conjugated to lipid nanoparticles composed of DOPE, cholesterol, and C14-PEG. The resulting cationic peptide-modified lipid nanoparticles were complexed with Cas9-encoding mRNA and sgRNA specific for the proprotein convertase subtilisin/kexin type 9 (PCSK9) gene. In a mouse model, intravenous administration of the lipoplexes was shown to lower serum levels of PCSK9 compared to treatment with empty carrier or a GFP-specific sgRNA/Cas9 mRNA lipoplex.

#### 2.1.2. Lipid Nanoshells

Although cationic lipid nanoparticles are promising systems for delivery of genome-editing components, complexation of cationic lipid nanoparticles with plasmid DNA or mRNA encoding Cas9 is hampered by certain limitations, such as poor stability in biological fluids [57,58,59]. To overcome stability issues posed by exposure of such cationic lipoplexes to serum, researchers have studied lipid shells encapsulating genome-editing cargo.

PEGylated lipid shells have been investigated for encapsulation of plasmid DNA encoding Cas9 and sgRNA specific for the Polo-like kinase I (PLK1) gene, encoding a key regulator of mitosis (Figure 2C) [33,60]. Condensed core materials for encapsulation into lipid shells were prepared by complexing plasmid DNA and chondroitin sulfate with protamine. The core complex was then encapsulated in a cationic lipid shell composed of DOTAP, DOPE, and cholesterol, and then further surface-modified with PEGylated lipid to improve stability in the circulation. Intravenous injection of PEGylated lipid shells encapsulating plasmid DNA into A375 tumor-bearing mice was found to reduce expression of PLK1 in tumor tissues and provide greater antitumor effects compared to treatment with a lipoplex of plasmid DNA and the commercial agent, Lipofectamine 2000.

In another PLK1-targeting application, cationic lipid shells were studied for encapsulation of core nanomaterials composed of a gold nanoparticle, Cas9 protein, and plasmid DNA encoding sgRNA specific for the PLK1 gene (Figure 2D) [34]. To formulate core nanomaterials, these researchers first modified gold nanoparticles with human immunodeficiency virus-1–derived TAT peptide and mixed them with Cas9 protein and plasmid DNA encoding sgRNA. The presence of negatively charged plasmid DNA encoding sgRNA enabled encapsulation of the core complex with a PEGylated lipid shell containing DOTAP and DOPE. Upon administration into a tumor-bearing mouse model, lipid nanoshells containing genome-editing components suppressed expression of PLK1 protein and exerted anticancer effects.

Mesoporous silica nanoparticles have also been studied as a core material for encapsulation into lipid nanoshells [35]. In this study, mesoporous silica nanoparticles were loaded with pCas9 and three different sgRNAs specific for PCSK9, apolipoprotein C (APOC3), and angiopoietin like 3 (ANGPTL3). The core nanoparticles loaded with genome-editing components were coated with cationic lipid shells composed of DOTAP, DOPE, cholesterol, and DSPE-PEG. Intravenous administration of the resulting genome editing component-loaded lipid shells was found to reduce serum triglycerides and cholesterol levels in a mouse model. Notably, cholesterol levels were significantly lower following treatment with all three sgRNA compared with treatment with any individual sgRNA.

### 2.2. Polymer-Based Delivery Systems

**Figure 3:**
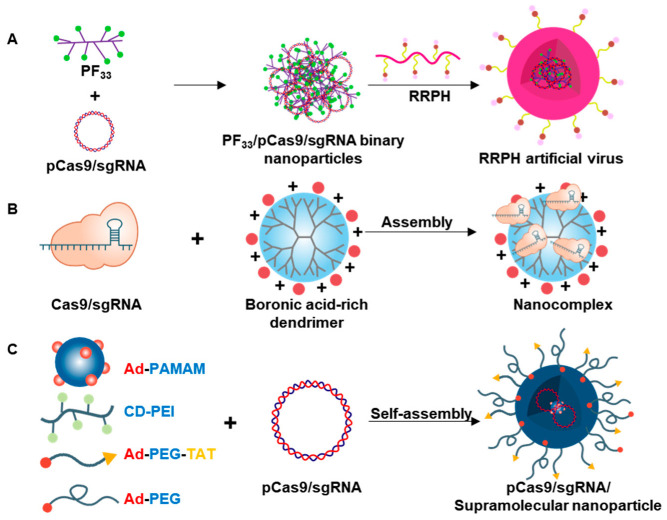

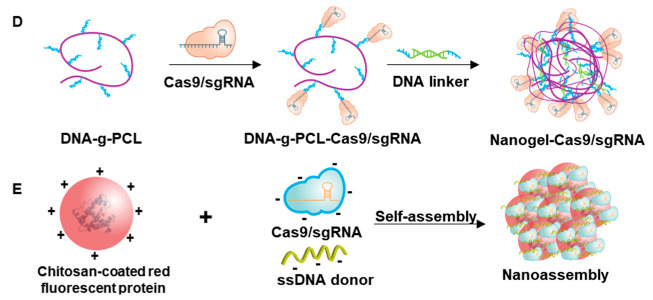
Polymeric systems for CRISPR/Cas9 and sgRNA delivery. (**A**) Fluorinated polyethyleneimine-based artificial virus for pCas9/sgRNA delivery. Adapted from [36], ACS, 2016. (**B**) Boronic acid-rich dendrimer for Cas9/sgRNA delivery. Adapted from [63], AAAS, 2019. (**C**) Supramolecular nanoparticle for pCas9/sgRNA delivery. Adapted from [38], WILEY-VCH, 2020. (**D**) Nucleic acid nanogel for Cas9/sgRNA delivery. Adapted from [64], RSC, 2019 (**E**) Chitosan-coated red fluorescent protein for Cas9/sgRNA and DNA donor delivery. Adapted from [65], RSC, 2019.

In addition to lipid vesicles, functional polymers have been widely studied for the delivery of Cas9 gene-editing components. The design of polymeric delivery systems depends on the form of Cas9: plasmid DNA, mRNA, or protein. For delivery of Cas9 in plasmid DNA or mRNA, the vectors are usually cationic, allowing their positive charges to complex with negatively charged cargoes. These charge-charge interactions readily allow formation of polyplexes between cationic polymers and nucleic acid genome-editing components. Polyplexes can protect nucleic acid cargoes from nuclease-mediated degradation through steric hindrance and increase cellular-uptake efficiency [61]. For delivery of Cas9 in protein form, the polymer needs to bear various functional groups that provide a strong interaction with Cas9. In general, complexes of Cas9 protein with sgRNA are reported to have an overall negative charge [62]. Representative polymer-based nanovesicles are illustrated in Figure 3.

#### 2.2.1. Polyethylenimine

Polyethyleneimine (PEI), which consists of amine groups and repeated two-carbon (CH_2_-CH_2_) spacers, has been studied for delivery of various nucleic acid-based gene therapeutics. Depending on its structure, PEI is classified as linear or branched type. In linear PEI, secondary amines are the only groups responsible for cationic charge. However, in branched PEI, heterogeneous amines, including primary, secondary, and tertiary amines, also contribute to cationic charges. Compared with linear PEI, the branched type is known to provide a smaller-sized polyplex with higher condensation efficiency, but suffers from more notable toxicity [66]. Among various sizes, 25 kDa PEI in linear or branched forms has been widely used for both in vitro and in vivo delivery. PEI of various sizes and with different modifications has been studied for delivery of genome-editing components.

A recent study applied amphiphilic PEI derivatives to deliver plasmid DNA encoding Cas9 and two sgRNAs specific for programmed death-ligand 1 (PD-L1) [67]. PEI 600 Da, an amphiphilic PEI derivative, was modified with hydrophobic stearic acid, then complexed with plasmid DNA and coated with human serum albumin. The resulting albumin-coated polyplexes were shown to decrease expression of the PD-L1 gene in CT26 colorectal cancer cells, reducing PD-L1 protein levels by more than 6-fold compared to treatment with a complex of Lipofectamine and genome-editing components. However, this study did not compare toxicity or gene-editing efficiency with unmodified PEI and did not evaluate the gene-editing performance of this system in an animal model. Careful assessment in further studies will be required before translation of this material for human use.

Another study has reported a PEI derivative, created by crosslinking branched PEI 600 Da with β-cyclodextrin (β-CD), for delivery of pCas9 and two types of sgRNA specific for hemoglobin subunit beta and rhomboid 5 homolog 1 (RHBDF1) [68]. At an N/P ratio (molar ratio of nitrogen in PEI to phosphate in DNA) of 60, pCas9 was completely condensed with β-CD–crosslinked PEI, forming 200-nm polyplex particles. Compared with unmodified 600 Da PEI, β-CD–crosslinked PEI was shown to enhance delivery efficacy while reducing associated toxicity. Introduction of polyplexes of β-CD–crosslinked PEI into HeLa cells knocked down the target genes, hemoglobin subunit beta and RHBDF1, with efficiencies of 19.1% and 7%, respectively. By comparison, treatment with 25 kDa PEI-based polyplexes showed hemoglobin subunit beta- and RHBDF1-knockdown efficiencies of 2.8% and 0%, respectively. Since β-thalassemia is a genetic disorder associated with a mutation in the hemoglobin subunit beta gene [69], β-CD–crosslinked PEI was suggested as a delivery system for genome editing-based therapy of β-thalassemia.

Fluorine modifications have been exploited for changing the physical and biological properties of small molecules or macromolecules [70,71]. Fluorination can increase the stability of proteins and enhance the therapeutic efficacy of drugs. Applied to polymer chemistry, fluorination can affect interactions of modified polymers with cellular membranes. Fluorinated materials have been reported to increase the binding affinity for cell membranes and promote endosome escape [72]. In addition, fluorinated polymers feature an extreme low surface energy, which assists polymer aggregation even at low concentrations [73]. Another benefit of fluorinated polymers is their ability to condense genetic materials at low N/P ratios.

Fluorinated PEI has been studied for the delivery of genome-editing components (Figure 3A) [36]. In this study, 1.8kDa PEI was fluorinated by conjugation with heptafluorobutyric anhydride, and the resulting fluorinated PEI 1.8 kDa was complexed with plasmid DNA encoding Cas9 and sgRNA. The included sgRNA targeted the MutT homolog 1 (MTH1) gene, encoding a protein that interrupts a natural pathway that protects cancer cells from apoptosis [74]. The polyplex of gene-editing components and fluorinated PEI was further coated with PEGylated hyaluronic acid and the cell-penetrating peptide, RGD-RRRRRRRR. The peptide- and hyaluronic acid-modified polyplex provided 1.2-fold higher knockout efficiency of the target gene MTH1 compared with the commercial transfection agent Lipofectamine 3000, and exerted tumor-suppressing activity that correlated with reduced expression of MTH1 in tumor tissue.

#### 2.2.2. Polyamidoamine

Polyamidoamine (PAMAM) is a highly branched polymer that is popular for use as a vector for gene delivery. However, the use of PAMAM dendrimers has been hampered by their considerable toxicity. To reduce toxicity and improve gene-delivery efficiency, Abedi-Gaballu et al. developed various derivatives of PAMAM [75]. Modification of PAMAM with phenylboric acid to increase the capacity for carrying Cas9 protein has also been recently reported (Figure 3B) [63]. Unmodified PAMAM bears only amine groups suitable for interaction with negatively charged plasmid DNA and thus lacks the ability to interact with protein. Unlike plain PAMAM, phenylboric acid-modified PAMAM was shown to provide various driving forces for enhanced interactions with protein. These interactions include coordination between boronic acid and cationic amine or imidazole of proteins, π-π interactions of aromatic rings, and ionic interactions with carboxylic and cationic species on proteins. The interaction of boronic acid-PAMAM with Cas9 protein and sgRNA specific for *GFP* yielded a nanoparticle with a size of 300 nm. The knockout efficiency of the polyplex was 40% in HEK293T-GFP cells, whereas that of conventional CRISPRMAX, whereas that of control CRISPRMAX vector (Invitrogen, USA), a commercially available cationic lipid-based vector for Cas9 protein delivery, was slightly higher than 20%. These results suggest the potential application of this system for in vivo gene editing. However, the fate of the boric acid derivative and its metabolic products in the body needs to be assessed from the standpoint of long-term toxicity before this system can be translated for human use.

In another study, PAMAM was grafted with adamantane (Ad-PAMAM) to form a dendrimer building block (Figure 3C) [38]. Beta-cyclodextrin (β-CD) is a cyclic oligosaccharide composing of seven glucopyranose units. β-CD forms a cone-shaped structure containing hydrophobic pocket. The inclusion of adamantane in the hydrophobic pockets of β-CD allowed adamatane-PAMAM and adamantane-grafted PEG to crosslink with β-CD-conjugated PEI, producing polymeric nanoparticles. The nanoparticle was further modified with TAT peptide to facilitate cellular uptake. Complexes of TAT peptide-modified Ad-PAMAM particle with plasmid DNA encoding Cas9, sgRNA specific for GFP, and plasmid DNA encoding GFP were shown to provide efficient GFP knock-in in vivo. After intravitreal injection of polyplexes into the eyes of mice, GFP signals were persistently observed in the retina from day 18 to day 30 post dose.

These results highlight the potential future use of this polyplex system to correct mutated genes in genetic ocular diseases. However, the combined use of cationic PEI and PAMAM in the same carrier can increase the toxicity of the carrier. Thus, future studies are needed to study the side effects of the carrier on retinal integrity and visual ability before translation for human use can be guaranteed.

#### 2.2.3. Other Synthetic Polymers

Other synthetic polymers have been studied for delivery of genome-editing components in addition PEI and PAMAM, including metal-coordinated polymers and neutral polymers. Although a high charge density on the polymer backbone is a critical factor for ensuring high gene-loading capacity, subsequent toxicity is a major concern. Thus, there remains a need for low-toxicity cationic polymers or neutral polymers for delivery of gene-editing tools. Several efforts have been made to minimize possible toxicity using low-charge-density quaternaryamine polymers or neutrally charged polymers.

In one such application, a nanomicelle was constructed using a quaternary amine polymer derived from trimethyl amine-grafted poly(propylene oxide) for delivery of plasmid DNA encoding Cas9 and sgRNA [39]. A cationic quaternary amine moiety was introduced for interaction with plasmid DNA, and the quaternary amine polymer and plasmid DNA polyplex was further stabilized by inclusion of the co-block polymer, poloxamer F127. The sgRNA included for genome editing was designed to be specific for HPV18 E7, an oncogene known to induce uncontrolled cell proliferation. In HeLa tumor-bearing mice, intratumoral injection of the polyplexes resulted in more than a 60% reduction in tumor volume.

In a recent study, a noncationic DNA-grafted polymer was used for delivery of Cas9 ribonucleoprotein and sgRNA specific for GFP (Figure 3D) [64]. A polycaprolactone polymer backbone was grafted with oligoDNA via click chemistry, yielding a multivalent DNA-grafted polymer. The sequence of the tethered oligoDNA was complementary to the tail of the guide RNA of the Cas9/sgRNA complex, enabling hybridization of the oligoDNA. A second oligoDNA linker was introduced to crosslink DNA and polycaprolactone, yielding a DNA-crosslinked polymeric nanogel containing Cas9/sgRNA loaded via a sequence-specific hybridization mechanism. The resulting Cas9/sgRNA complex-loaded nanogel was shown to reduce GFP signals by 21.1% and the indel value by 18.7%. Notably, this was the first study to use noncationic DNA-based material for delivery of genome-editing components. Additional studies on the safety and efficacy of this system at the animal level will be needed in the future.

#### 2.2.4. Chitosan

Chitosan, a ubiquitous component of the shells of shrimp and other crustaceans, is a natural cationic polysaccharide constructed of deacetylated units (D-glucosamine) and acetylated units (N-acetyl-D-glucosamine) bonded by β-1,4 glycosidic linkages. As it bears a number of primary amines, chitosan is positively charged at acidic pH through protonation of amine groups [76,77]. Chitosan has been studied as a biomaterial for applications such as tissue engineering, cosmetics, and drug delivery owing to its biocompatibility and biodegradability [78]. Chitosan can be degraded by lysosomal enzymes, producing nontoxic oligosaccharide and monosaccharide metabolites [79].

To date, only a few studies have reported the use of chitosan derivatives for delivery of genome-editing components. In one study, chitosan-coated negatively charged fluorescent protein was used for delivery of Cas9 protein and sgRNA (Figure 3E) [65]. For complexation with chitosan, Cas9 protein was modified with polyglutamic acid. Polyplexes composed of chitosan, red fluorescent protein, a Cas9 protein derivative, and sgRNA specific for glutathione peroxidase-4 (GPX4) were found to form a 177-nm nanoparticle. The knock-in efficiency of the GPX4 gene in the genome of HEK297 cells was reported to be 12.5% ± 3.0%. Additional studies will be needed to provide in vivo evidence of genome-editing component delivery. Moreover, potential immune stimulation by chitosan needs to be assessed to ensure that Cas9 protein delivered via chitosan does not result in an elevated immune response.

Various polymers have been investigated as vectors for delivery of gene-editing tools. Each polymer has its respective advantages and limitations that depend on structure, size, topology, and charge density. These parameters strongly influence the behavior as well as toxicity of polymer vectors in biological systems. Current studies have reported the feasibility of these polymers in genome-editing applications at the cell and/or animal level. For translation to human use, further studies will be needed to fine-tune the relevant parameters so as to achieve high efficiency with as little toxicity as possible. If these issues were addressed, polymers would be a powerful platform for delivery of genome-editing components for various incurable diseases.

### 2.3. Extracellular Vesicles

Cell membrane-derived vesicles play an important role in cell-to-cell communication and cellular material transfer. As knowledge in this field has increased, there has been a corresponding increase in studies investigating the use of extracellular vesicles as drug-delivery systems [80]. ‘Extracellular vesicle’, a term that is applied collectively to membrane-derived particles obtained from cells, are generally classified into the following three types according to their origin [81]: (1) exosomes, nano-sized vesicles derived from the endosomal pathway; (2) microvesicles, which directly bud from the plasma membrane; (3) apoptotic bodies, which are secreted from the surface of dying cells.

**Figure 4:**
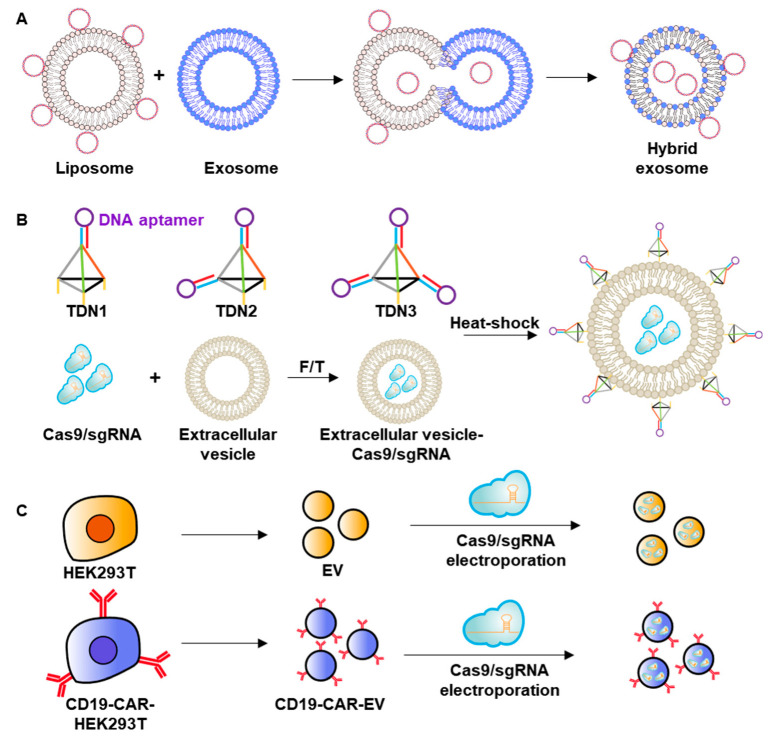
Extracellular vesicles for delivery of genome-editing components. (**A**) Exosome-liposome hybrid nanoparticles for delivery of plasmid DNA encoding Cas9 and sgRNA. Adapted from [86], WILEY-VCH, 2018. (**B**) DNA aptamer-conjugated extracellular vesicles for Cas9/sgRNA delivery. Adapted from [41], Oxford University Press, 2020. (**C**) Chimeric antigen receptor-extracellular vesicles for Cas9 ribonucleoprotein with complexed sgRNA. Adapted from [43], Elsevier, 2020.

Such cell-derived vesicles offer several advantages for application as delivery systems. First, because of their origin and intrinsic stability, extracellular vesicles are often less immunogenic and show reduced clearance by the reticuloendothelial system compared with synthetic vesicles such as liposomes [82,83]. In addition, as part of the endogenous material transport machinery of the body, extracellular vesicles can efficiently pass through the blood–brain barrier or plasma membranes via intrinsic trafficking mechanisms [84]. Vesicle membrane composition, which depends on the producing cells and the presence of certain membrane proteins, greatly affect the tropism or target selectivity of extracellular vesicles [85]. All of these features are prerequisites for designing an ideal delivery system for genome-editing components. Representative examples of extracellular nanovesicles are illustrated in Figure 4.

#### 2.3.1. Exosomes

Cancer cell-derived exosomes have been investigated as a delivery system for Cas9 protein and sgRNA for anticancer therapy [40]. Taking advantage of the tropism towards their cell of origin, exosomes produced from SKOV3 ovarian cancer cells, designed to contain Cas9 protein and sgRNA specific for poly (ADP-ribose) polymerase-1 (PARP‑1), were introduced by electroporation to knock out PARP‑1. Compared with exosomes derived from HEK293 cells, exosomes from SKOV3 cells showed enhanced uptake into SKOV3 recipient cells. Intravenously injected SKOV3-derived exosomes showed significant accumulation in tumor tissues of SKOV3-xenografted mice and resulted in significant PARP‑1 knockout, exhibiting 27% indel efficacy. As PARP‑1 is mainly associated with DNA repair processes, the authors of this study combined exosome treatment with cisplatin, which induces DNA damage. This combinational anticancer therapeutic regimen exerted synergistic anticancer efficacy, inhibiting cancer proliferation by 57% compared with 30% and 21.6% inhibition by exosome and cisplatin treatment alone, respectively.

To increase sgRNA and Cas9 protein loading into exosomes, researchers have applied the concept of specific interactions between antibody and antigen [87]. For this purpose, CD63, a member of the tetraspanin family that is mainly associated with intracellular vesicle membranes, was fused with GFP. Cas9 protein was also modified by conjugating with an anti-GFP antibody fragment of a single monomeric variable domain (nanobody). Consistent with predictions that specific interactions between GFP and the GFP nanobody would increase the entrapment of Cas9 in the exosome, GFP-fused exosomes were found to carry a significantly higher amount of sgRNA/Cas9 complex compared with plain exosomes. The increased amount of sgRNA and Cas9 protein loaded in the exosome led to improved genome-editing efficacy in an A549 cell line model.

In another attempt to enhance the loading of genome-editing components in exosomes, researchers developed an exosome-liposome hybrid system (Figure 4A) [86]. To increase the encapsulation efficiency of plasmid DNA, these researchers incubated exosomes derived from HEK293FT cells with plasmid DNA and liposome complexes. Subsequent fusion of the exosome and liposome allowed plasmid DNA to be encapsulated in the hybrid exosome. The hybrid exosome was found to significantly enhance the expression of GFP-encoding plasmid DNA in mesenchymal stem cells, resulting in 13.2% GFP-positive cells compared with 0.11% and 1.46% for cells treated with exosome or liposome alone, respectively. For delivery of genome-editing components, the exosome-producing cells were transfected with sgRNA specific for the runt-related transcription factor 2 or β‑catenin (CTNNB1) gene, and exosomes were collected and fused with lipoplexes containing pCas9. The indel percentage at the target gene was not determined and should be explored in the future.

The surfaces of exosomes have been modified for targeted delivery to specific cancer cell types (Figure 4B) [41]. In one example, the surfaces of HEK293T cell-derived exosomes were decorated with a cholesterol-tagged DNA aptamer. Cas9 protein complexed with an sgRNA specific for WNT10B, encoding a secreted member of the WNT family, was encapsulated in exosomes using a repeated freeze–thaw process and modified with TLS11a aptamer targeting HepG2 hepatocellular cancer cells. Aptamer-modified exosomes containing Cas9/sgRNA were found to exhibit enhanced binding to HepG2 cells, but not MCF-7 cells. In a HepG2 tumor-bearing mice model, intravenously injected TLS11a aptamer-tethered exosomes containing Cas9/sgRNA provided 5-fold higher WNT10B genome editing and greater anticancer efficacy against HepG2-derived tumors compared with treatment with plain exosomes.

#### 2.3.2. Microvesicles

Microvesicles, another type of extracellular vesicle, have gained attention as a delivery system for genome-editing components. Unlike exosomes, which are derived from the endosomal pathway, microvesicles are released directly from the cell membrane [88]. Compared with exosomes, which typically have a diameter of 30–150 nm, microvesicles are larger, ranging from 100 to 1000 nm [89], offering the advantage of greater cargo-loading capacity [90]. Moreover, since microvesicles are directly derived from the surface membrane, their membranes are closer in composition to those of the producing cell, whereas exosomes tend to more closely reflect the composition of intracellular endosomes.

The potential of microvesicles as delivery systems for genome-editing components has been investigated in a hepatocellular carcinoma model [42]. In this example, vesicles were collected from Cas9-expressing HepG2 or HEK293 cells and electroporated with plasmid DNA encoding sgRNA specific for the IQ-domain GTPase-activating protein 1 (IQGAP1) gene. Microvesicles from HepG2 and HEK293 cells, with sizes of 355.5 and 320.0 nm, respectively, showed a plasmid DNA loading capacity of ≈3.5%. The engineered microvesicles were found to induce genome editing in HepG2 cells, producing an indel frequency at the target gene of up to 19.5% Although microvesicles from HepG2 cells showed tropism towards intact HepG2 cells, the researchers found that cancer-associated mRNA and proteins delivered by these cancer cell-derived vesicles promoted cancer progression.

Direct engineering of membrane-producing cells is another approach for modifying the surface composition of membrane-derived vesicles. By transfecting a CD19-specific chimeric antigen receptor-encoding gene into HEK293T cells, Xu et al. established an engineered cell line stably expressing chimeric antigen receptor on the cell membrane (Figure 4C) [43]. The resulting engineered microvesicle was electroporated with plasmid DNA encoding Cas9 and sgRNA specific for the MYC oncogene. After intracardial injection into Raji tumor-bearing NOD/SCID mice, the engineered microvesicles showed higher distribution to tumor tissues and more effective MYC genome editing compared with plain exosomes.

Although extracellular vesicles have shown potential as delivery systems for genome-editing components, a number of challenges remain to be solved for clinical application. Even though tropism towards the cell of origin enhances accumulation of certain extracellular vesicles at their target site, most nanovesicles have broad selectivity for various tissues. It was previously shown that microvesicles generated from HEK293 cells, one of the most popular extracellular vesicle-producing cells, are broadly distributed throughout the body, an unexpected outcome given the intended tumor-targeting capability [42]. Therefore, because off-target effects are the most pressing concern with genome-editing technology, achieving precise targeting of specific cells while maintaining the inherent advantages of extracellular vesicles will be necessary for safe genome editing.

Ambiguities in the definition of extracellular vesicles also limit the clinical applications of this technology. Although it is possible to classify extracellular vesicles into three types according to their biogenesis pathway and particle size, unique markers that can define a specific subtype among various secreted vesicles are lacking. As most of the exosomes, microvesicles, and virus-like particles referred to above were isolated and purified using the same procedure, it will be necessary to develop a method for distinguishing or separating specific types of vesicles containing genome-editing tools. Moreover, quality criteria for internal biological components, in terms of consistency and quantity, should be established for clinical applications.

Few studies have investigated apoptotic bodies as potential extracellular vesicles. Apoptotic bodies differ from exosomes and microvesicles in that they are derived from dying cells. During the process of apoptosis, cells release vesicles ranging from 50 to 5000 nm in size that include shrunken cell debris and fragments of DNA or cellular organelles. The ability to transmit a death-promoting message to recipients by regulating inflammation and immune responses—a unique feature of the apoptotic body—may have the potential to cause side effects when delivered to normal cells. In addition, the cellular engineering techniques used for isolating exosomes and microvesicles cannot be applied to collect apoptotic bodies from dying cells, and techniques for preparing apoptotic bodies are not yet fully developed.

### 2.4. Peptide/Protein-Based Systems

**Figure 5:**
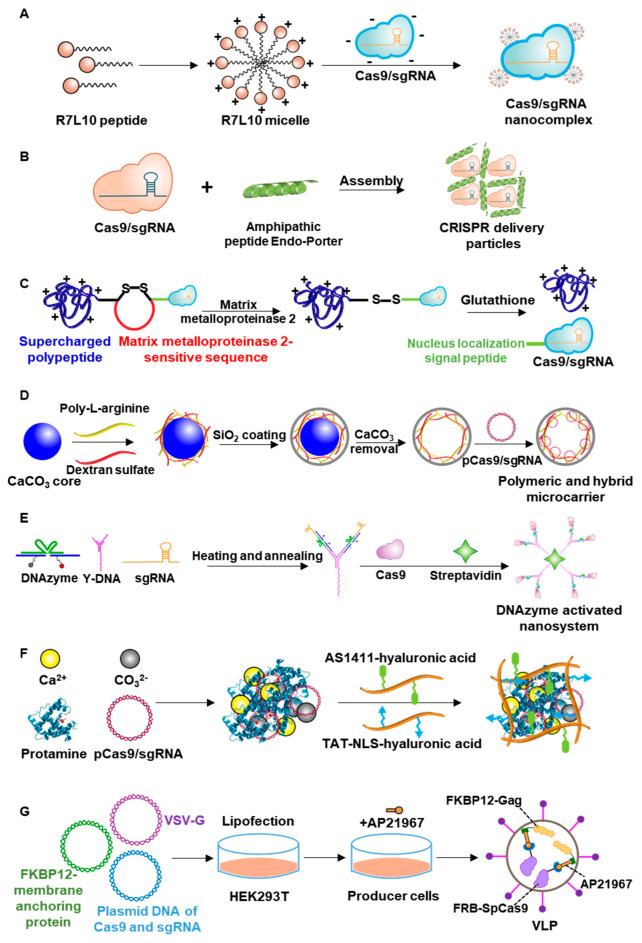
Peptide/protein-based systems for delivery of genome-editing components. (**A**) R7L10-based micelle for delivery of Cas9 ribonucleoprotein (RNP) complexed with sgRNA. Adapted from [44]. Nature Publishing Group, 2019 (**B**) Amphipathic peptide-based nanocomplexes for delivery of Cas9 ribonucleoprotein complexed with sgRNA. Adapted from [45], ASBMB, 2018. (**C**) Supercharged polypeptide-mediated nanocomplexes for delivery of Cas9 and sgRNA complex [98], ACS, 2019. (**D**) Poly-L-arginine-based polymeric nanoparticles for delivery of plasmid DNA encoding Cas9 and sgRNA. Adapted from [99], Elsevier, 2017. (**E**) Streptavidin and DNAzyme-based nanoassembly for delivery of Cas9 ribonucleoprotein complexed with sgRNA. Adapted from [100], RSC, 2019. (**F**) Protamine-based nanocomplexes for delivery of plasmid DNA encoding Cas9 and sgRNA. Adapted from [101]. (**G**) Cas9/sgRNA-loaded extracellular vesicles for genomic exon skipping. Adapted from [48], Nature Publishing Group, 2020.

Proteins and peptides have attracted considerable attention as delivery systems for genome-editing components, at least in part, because of their advantages in terms of biocompatibility and biodegradability [91,92]. Various types of peptides, including α-helical and β-sheet peptides, cyclic and linear peptides and amphiphilic peptides, have been studied for the delivery of long and short RNA, plasmid DNA, and Cas9/sgRNA ribonucleoproteins [93,94,95]. For protein-based delivery systems, positively charged proteins provide the ability to condense nucleic acids, which enhances the efficiency of gene transfection [96]. Surface modification of peptide and protein-based systems with targeting moieties can improve the delivery of genome-editing components to specific sites [97]. Representative examples of peptide/protein-based delivery systems for genome-editing components are illustrated in Figure 5**.**

#### 2.4.1. Peptides

The amphiphilic R7L10 peptide was designed to complex with Cas9/gRNA ribonucleoproteins for neuronal gene editing in Alzheimer’s disease (Figure 5A) [44]. In this example, nanocomplexes were prepared by exploiting electrostatic interactions between the positive charge of the R7L10 peptide (NH2-RRRRRRRLLLLLLLLLL-COOH) and negative charge of the Cas9/sgRNA ribonucleoprotein. Accumulation of amyloid β protein in Alzheimer’s disease is known to be regulated by β-secretase 1 [102]. In the 5XFAD mouse model of Alzheimer’s disease, Cas9/sgRNA complexed with peptide micelles was found to reduce the expression of β-secretase 1 in the hippocampus and decrease amyloid β protein secretion compared with controls.

An amphipathic α-helical peptide consisting of leucine and histidine residues has been used for delivery of Cas9/sgRNA ribonucleoprotein to adipose cells (Figure 5B) [45]. Peptide and Cas9/sgRNA ribonucleoproteins were complexed based on charge-charge interactions. Upon treatment of white adipocytes, these nanocomplexes were shown to edit the nuclear receptor-interacting protein 1 gene, as evidenced by a mutation frequency of 43.8% in the target genomic locus. This study is noteworthy for its assessment of the feasibility of gene editing in adipocytes, which carries the potential of treating metabolic disease.

The cell-penetrating peptide (CPP), GGGGRRRRRRRRRLLL, has been studied for delivery of Cas9 protein and sgRNA [103]. CPPs are positively charged, short (generally 5–30 amino acids) peptides that are known to penetrate the cell membrane [104]. Kim and colleagues conjugated Cas9 protein to the indicated CPP via a thioether bond and complexed sgRNA through electrostatic interactions. The resulting nanocomplexes showed mutations at the CCR5 genomic locus when tested in several cell types, including HEK293T cells (16.0%), HeLa cells (5.5%), and NCCIT cells (2.7%), with no detectable off-target effects.

Another membrane-permeating peptide, termed supercharged peptide (KKKKPLFGLFFGLF), has been investigated for delivery of tumor microenvironment-responsive Cas9/sgRNA complexes (Figure 5C) [98]. In this example, a matrix metalloproteinase 2-responsive sequence was introduced between Cas9 protein and the supercharged peptide as a dithiocyclopeptide linker containing a disulfide bond. In addition, a nuclear-localization signal peptide was inserted between Cas9 protein and the linker. The system was designed such that matrix metalloproteinase 2 in the tumor microenvironment cleaves the dithiocyclopeptide sequence, liberating a fusion protein composed of the Cas9 ribonucleoprotein, nuclear-localization signal peptide, and supercharged peptide. The resulting peptide complexes were shown to induce indels at the CCR5 genomic locus with a frequency of 13.4%, 31.9%, and 28.3% in L02 normal human liver cells, HeLa cervical cancer cells and A549 epithelial carcinoma cells, respectively. Further studies are needed to explore the efficacy of CCR5 genome editing by this peptide complex in animal models.

Poly-_L_-arginine, a positively charged polypeptide, has been studied for delivery of plasmid DNA encoding Cas9 and sgRNA specific for the dTomato fluorescent protein gene (Figure 5D) [99]. In this study, poly-_L_-arginine and dextran sulfate were used to prepare capsules via layer-by-layer assembly on CaCO_3_ particles. A hollow structure was then fabricated by removing CaCO_3_ particles using ethylenediaminetetraacetic acid and coated with SiO_2_. Plasmid DNA encoding Cas9 and sgRNA was complexed to the hollow particles by electrostatic interaction. Compared with the commercial transfection reagent Metafectene PRO, these microcapsules provided 1.8-fold higher transfection efficiency. After delivery of plasmid DNA encoding Cas9 and dTomato-specific sgRNA using the microcapsule, 32% of dTomato-expressing cells lost their red fluorescence, whereas only 12% of cells in the Metafectene PRO group lost their fluorescence. These findings remain to be validated in vivo.

#### 2.4.2. Proteins

A streptavidin and DNA linker-based system has been studied for DNAzyme-controlled delivery of Cas9 protein and sgRNA (Figure 5E) [100]. In this study, double-stranded DNAzymes were hybridized with the two ends of Y-shaped DNA and partially hybridized with sgRNA. The other end of the Y-shaped DNA was conjugated with a biotin molecule that could be conjugated with streptavidin. Cas9 protein was then loaded onto the resulting nanoassembly through interactions with sgRNA. In living cells, the presence of Mn^2+^ in the cytoplasm was expected to cleave the DNAzyme, releasing the Cas9/sgRNA complex. Delivery of Cas9 and *GFP*-specific sgRNA using this system was shown to decrease GFP fluorescence compared with untreated cells. Although this is an elegantly designed system, nonspecific degradation of DNA in vivo may be a challenge for further development.

Aptamers based on protamine, an arginine-rich protein used for condensing and stabilizing DNA [105], have been studied for delivery of plasmid DNA encoding Cas9 and sgRNA specific for CTNNB1 (Figure 5F) [101]. In this study, Cheng and colleagues constructed a CaCO_3_ particle containing a protamine core, and complexed it with plasmid DNA through electrostatic interactions. The core was then coated with AS1411 aptamer-conjugated hyaluronic acid and TAT peptide-conjugated hyaluronic acid by exploiting electrostatic interactions between the negative charge of hyaluronic acid and positive charges of protamine and calcium. Treatment of H1299 cells with protamine-based complexes was shown to provide a gene-editing efficiency of 40.2%.

#### 2.4.3. Virus-Like Particles

Virus-like particles (VLPs) are engineered protein particles that form a viral-like structure. After completing the viral replication pathway, viral structural proteins expressed in the cell self-assemble into particles that lack a pathogenic viral genome. Therapeutic cargos can be entrapped within VLPs during self-assembly of particles [106]. Aided by their viral origin, VLPs offer high transduction efficiency, making them a promising tool for delivery of genome-editing components [107].

VLPs derived from vesicular stomatitis virus (VSV) have been studied for delivery of Cas9 protein and sgRNA specific for EGFP [46]. This study utilized VLPs composed of fusogenic VSV glycoprotein so as to minimize viral elements such as Gag. Cas9/sgRNA ribonucleoproteins were entrapped inside VLPs during the assembly process. As sgRNA delivered in plasmid DNA requires a multistep expression process in the nucleus and translocation to the cytoplasm, its efficacy is sensitive to transfection efficiency, which is low for VLPs. This potential limitation was overcome by introducing a T7 RNA polymerase transcription system into VLPs that produces sgRNA directly in the cytosol rather than in the nucleus. VLPs containing Cas9 and *EGFP*-specific sgRNA induced a 50% knockdown of EGFP fluorescence in EGFP-expressing HEK293 cells. In EGFP transgenic mice, intracardiac injection of VLPs containing genome-editing components generated a larger population of non-fluorescent cardiomyocytes compared with VLPs containing scrambled sgRNA.

VLPs derived from murine leukemia virus have been studied for delivery of genome- components to primary cells [47]. Murine leukemia virus-mimicking VLPs were engineered to contain VSV glycoprotein and baboon retroviral envelope glycoprotein to improve transduction efficiency in primary cells [47]. Cas9 protein was conjugated to VLPs through a cleavable linker, and sgRNA was complexed with Cas9 protein. Injection of VLPs containing a tyrosinase (TYR)-specific sgRNA into mouse zygotes was found to disrupt the TYR gene; notably, this disruption was transmitted to offspring, generating albino mice. Moreover, retroorbital injection of VLPs containing sgRNA specific for hydroxyphenylpyruvate dioxygenase (HPD) in a Fah^−/−^;Rag2^−/−^;Il2rg^−/−^ mouse model was found to induce a detectable level (≈13%) of gene editing. Although this study is preliminary, it suggests a potential genome-editing system for treatment of hereditary tyrosinemia type I.

Instead of direct conjugation of Cas9 protein to a structural protein associated with VLP formation, an alternative strategy for Cas9 protein loading is chemical-induced interaction. This approach exploits the rapamycin property of dual binding to FK506-binding protein (FKBP)-12 and FKBP-rapamycin binding domain (FRB), which induces dimerization of FKBP-12 and FRB. This property was applied to binding of FKBP-12-tagged HIV Gag protein and FRB-tagged Cas9 protein during VLP packaging (Figure 5G) [48]. Cas9 protein was effectively packaged in the VLP, as evidenced by the enhanced genome-editing efficacy in the presence of rapamycin. Notably, the engineered VLPs were shown to induce genome editing in various hard-to-transfect cells. In a Duchenne muscular dystrophy mice model, intramuscular injection of VLPs containing sgRNA specific for dystrophin produced a genomic deletion frequency of 1.1%.

Among the limitations of VLP-mediated delivery is the specificity of binding to target cells, which will need to be improved. As an example, VSV glycoprotein present on VSV-derived VLPs can bind to the low-density-lipoprotein receptor, allowing it to enter various cell types [108]. Specific binding and delivery would be important in minimizing potentially harmful side effects of off-target genome editing in translational studies.

## 3. Targeting Ligands for Genome Editing

The targeting concept has been applied to nanotechnology as a strategy for increasing the therapeutic efficacy of drugs and reducing their side effects [109]. With physiological barriers and molecular properties of target cells in mind, researchers have further engineered drug-delivery systems to endow their therapeutic cargoes with higher selectivity; considerable progress has been made in this area, especially in cancer therapy [110]. The concept of directed targeting has recently been applied to delivery of genome-editing components as a strategy for overcoming low efficiency and off-target effects. A number of different types of chemical or biological ligands have been investigated for this purpose (Table 2).

### 3.1. Chemical Ligands for Targeted Delivery

A number of different chemical ligands have been investigated as targeting moieties. Among them, galactose has been used as a ligand for targeted drug delivery to asialoglycoprotein (ASGP) receptor-expressing hepatocytes. Various types of drug-carrying liposomes and polymers have been modified with galactose for liver targeting [119,120]. A recent study reported the development of a triple-targeting delivery system employing galactose, TAT, and a nuclear-localization signal for delivery of genome-editing components [111]. In this study, TAT peptide-conjugated gold nanoclusters were complexed with sgRNA and nuclear-localization signal-tagged Cas9 protein, and the resulting gold nanocluster-based complex was then encapsulated with galactose-modified lipid layers composed of DOTAP, DOPE, cholesterol, and galactose-conjugated PEG-DSPE. Surface-displayed galactose served to target the ASGP receptor on hepatocytes, and TAT and nuclear-localization signal served to enhance delivery to the nucleus of target cells. Intravenous administration of the galactose-modified system carrying a sgRNA specific for the PCSK9 gene, encoding a key player in cholesterol metabolism, was found to disrupt the target gene and reduce low-density lipoprotein cholesterol by 30% compared with saline-treated controls.

In a related application, triantennary N-acetylgalactosamine was conjugated with therapeutic oligonucleotides for ASGP receptor-mediated hepatocyte targeting [121]. A more recent study adapted this approach for targeted delivery of genome-editing components, tethering N-acetylgalactosamine to a Cas9/sgRNA ribonucleoprotein via a disulfide bond [122]. The resulting trimeric ligand showed increased binding affinity for ASGP receptor, exhibiting a K_d_ value < 100 pM. In the HepG2 hepatocyte cell line, N-acetylgalactosamine–bound Cas9/sgRNA ribonucleoprotein showed ≈7-fold higher cellular uptake and 10-fold greater genome-editing efficiency compared with unmodified Cas9/sgRNA ribonucleoprotein.

Another chemical ligand that has been investigated for targeted delivery of genome-editing components is all-trans retinoic acid (ATRA) [112], which was previously reported to bind interphotoreceptor retinoid-binding protein and be selectively taken up by the retinal pigment epithelium. In this study, the surfaces of polymeric nanocapsules carrying Cas9 protein and sgRNA specific for a stop sequence that prevents expression of the tdTomato fluorescent protein gene were modified with all-trans retinoic acid. Subretinal injection of this all-trans retinoic acid-decorated polymeric nanocapsule into STOP-tdTomato transgenic Ail4 mice produced the predicted editing of the stop sequence, resulting in an increase in the tdTomato fluorescence-positive area around the retinal pigment epithelium compared with that observed following injection of plain nanocapsules.

Folate is another chemical ligand that is widely used in targeted delivery systems, reflecting the frequent overexpression of folate receptors on various cancers. In a recent study, folate-conjugated liposomes encapsulating plasmid DNA encoding Cas9 and sgRNA were developed for use in ovarian cancer genome editing [113]. Liposomes were constructed by adding folate-conjugated PEG-succinyl-cholesterol at 5 mole % to DOTAP and cholesterol lipid components, after which folate-conjugated cationic liposomes were complexed with plasmid DNA encoding Cas9 and sgRNA specific for DNA methylase I. Following intraperitoneal injection into SKOV-3 xenograft mice, these folate-modified lipoplexes exerted antitumor efficacy comparable to that of paclitaxel at a dose of 10 mg/kg.

### 3.2. Peptide Ligands for Targeted Delivery

Among various peptide-based ligands, the RGD peptide has been the most widely studied for use in delivering genome-editing components. Due to the strong affinity of RGD for integrin receptors expressed on numerous cancer cells, RGD peptide-tethered delivery systems have been used for cancer therapy. In one study, RGD peptide conjugation facilitated internalization of a tandem peptide-based nanocomplex system into multiple cell lines [114]. In this application, a tandem peptide composed of a CPP sequence and RGD sequence was constructed and its N-terminal was conjugated with particles packaging a palmitoyl lipid tail. Cas9/sgRNA ribonucleoproteins were entrapped during self-assembly of the tandem peptide-lipid conjugate. The resulting RGD peptide-modified nanocomplexes were shown to produce a target gene knockdown efficiency of 28% in HeLa cells, whereas plain nanocomplexes exerted no knockdown effect.

Core-shell structured liposome-templated hydrogel nanoparticles are another example of how RGD-mediated integrin receptor targeting can improve genome-editing efficacy [115]. In this example, polymeric hydrogels were formed through host–guest interactions by simply mixing β-CD-PEI and adamantine-engrafted PEI; CRISPR/Cas9 components, including sgRNA targeting PLK1, were subsequently efficiently loaded inside the hydrogel. The RGD peptide modification increased nanoparticle accumulation in tumor tissue by 2.6-fold compared with RGD-free nanoparticles following intravenous injection in U87 tumor-bearing mice. Subsequent genome editing of PLK1 led to tumor growth suppression, reducing tumor volume by 23.5% compared with the saline-treated control group.

Octa-arginine and reverse RGD peptide (DGR) have also been used for surface modification of cationic liposomes [123]. In this study, the surfaces of DOTAP-based cationic liposomes were modified with a peptide consisting of octa-arginine (R8) and DGR, and the resulting modified liposomes were complexed with plasmid DNA encoding Cas9 and sgRNA specific for hypoxia-inducible factor-1α (HIF1A). Surface modification with the peptide was found to increase the delivery of lipoplexes to BxPC-3 cells expressing integrin αvβ3 and neuropillin-1.

In addition to single-integrin targeting, dual-receptor targeting has been employed as a delivery strategy [36]. In this study, RGD and hyaluronan polymer were used for integrin αvβ3 and CD44 receptor targeting, respectively. Specifically, plasmid DNA encoding Cas9 and sgRNA specific for the MTH1 gene was incorporated into a core-shell through binding to the fluorinated polymer core, and the core was coated with hyaluronan polymer and R8-RGD peptide. Upon intravenous injection into an SKOV3 xenograft mouse model, the dual ligand-modified system showed significantly higher tumor accumulation and more effective MTH1 gene disruption than the respective mono ligand-modified delivery systems.

### 3.3. Antibodies and Aptamers for Targeted Delivery

In addition to chemical ligands, antibodies have received considerable research attention as targeted delivery ligands. Antibodies or aptamers are advantageous for this purpose because of their intrinsic ability to selectively recognize target antigens with high affinity. In one study, an antibody against ICAM1 (intercellular adhesion molecule 1) was conjugated to the surface of lipid and alginate-based nanogels entrapping plasmid DNA encoding Cas9 and sgRNA. In MDA-MB-231 tumor-bearing mice, intravenously injected antibody-modified nanolipogel showed 1.7-fold higher tumor accumulation and significantly higher genome-editing efficiency than plain nanolipogel.

In addition to antibodies, aptamers are another type of biomolecule that specifically binds target molecules with high affinity. Aptamers, which can be designed as short single-stranded DNAs, RNAs or peptides, interact with their targets through distinct three-dimensional structures [124]. Given their high binding affinity, low immunogenicity, and simple manufacturing process compared with antibodies, aptamers are suitable targeting moieties for drug-delivery systems [125]. Aptamer-mediated cell-specific targeting has also been investigated for the delivery of genome-editing tools.

Aptamers specific for nucleolin, which is overexpressed on various cancer cells, have been studied for targeted delivery of genome-editing systems [116]. In this study, protamine-based cationic nanoparticles were complexed with AS1411-functionalized carboxymethyl chitosan for delivery of plasmid DNA encoding Cas9 and sgRNA specific for CTNNB1 [116]. Introduction of AS1411 aptamer-modified delivery systems into HeLa cells was found to induce CTNNB1 genome editing (efficiency, 28.4%) and reduce β-catenin expression.

In another study, a delivery system was modified to contain dual nucleolin and mucin 1 aptamers [117], a strategy based on the fact that both nucleolin and mucin 1 are overexpressed on various cancer cell membranes. In this study, the delivery system was prepared by coprecipitating protamine in complex with plasmid DNA encoding Cas9 and sgRNA specific for focal adhesion kinase and incorporating AS1411 aptamer-conjugated heparin and mucin 1 aptamer-conjugated heparin through charge-charge interactions. MCF-7 breast cancer cells were used as an in vitro model. Due to the presence of high levels of nucleolin and mucin 1 on the cell surface, the dual aptamer-modified protamine nanocomplex showed enhanced cellular uptake and efficient genome editing, reducing expression of focal adhesion kinase by 50% compared with untreated cells.

In another study conducted by Liang and colleagues, an osteosarcoma-specific aptamer was studied as a targeting ligand for lipopolymer-mediated delivery of genome-editing components [118]. Using the cell-SELEX technique to screen for selective binders, these researchers identified the aptamer LC09 as showing high binding affinity for osteosarcoma cells. LC09 aptamers were conjugated to lipopolymer nanoparticles composed of branched PEI, PEG, and cholesterol, and then complexed to plasmid DNA encoding Cas9 and sgRNA. In a mouse model of orthotopic osteosarcoma, intravenously injected LC09-modified lipopolymer complexes showed increased accumulation in the tumor and enhanced genome editing compared with unmodified lipopolymer complexes.

## 4. Delivery Strategies for Genome Editing

**Figure 6:**
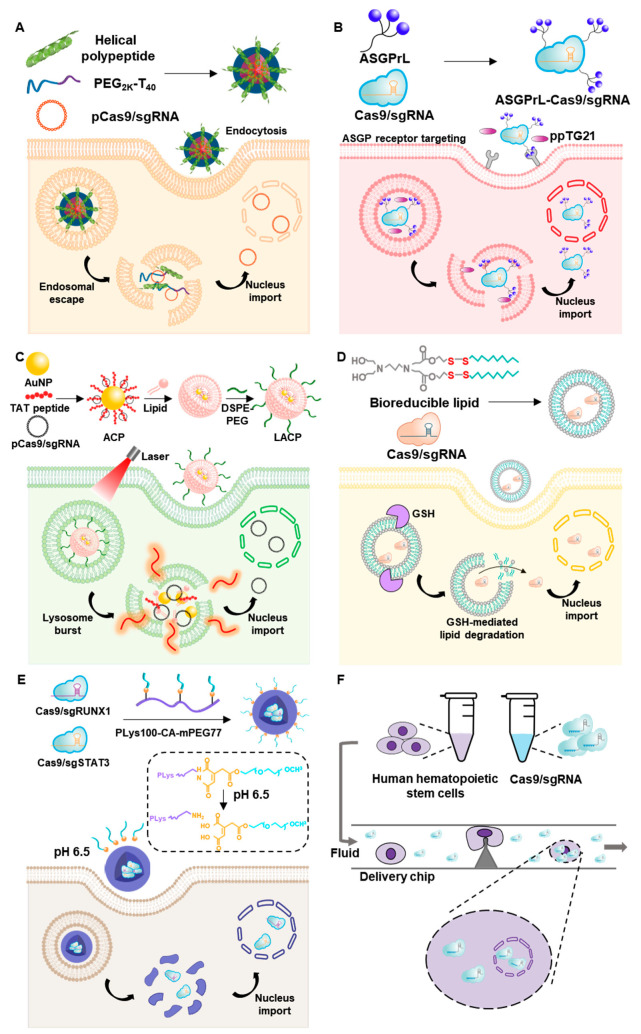
Delivery strategies for CRISPR/Cas9 and sgRNA. (**A**) Helical peptide-mediated endosomal escape. Adapted from [126], PNAS, 2018. (**B**) ASGP receptor targeting nanocomplexes for Cas9/sgRNA RNP delivery. Adapted from [122], ACS, 2018. (**C**) Photo-responsive liposome for pCas9 delivery. Adapted from [127], WILEY-VCH, 2018. (**D**) GSH-responsive lipid-containing liposome for Cas9/sgRNA RNP delivery. Adapted from [128], PNAS, 2016. (**E**) pH-responsive polymeric nanoparticle for Cas9/sgRNA RNP delivery. Adapted from [129], ACS, 2019. (**F**) Physical delivery of Cas9/sgRNA RNP using a microfluidic chip system. Adapted from [130], RSC, 2017.

Despite the wide range of materials that have been actively studied for drug-delivery systems, low delivery efficiency and off-target effects still remain as problems for nonviral delivery systems. Researchers have attempted various strategies for overcoming these limitations, including facilitation of endosomal escape, stimulus-responsive release, and physical delivery of genome-editing components (Figure 6, Table 3).

### 4.1. Chemical/Molecular Delivery Strategies

#### 4.1.1. Chemical/Peptide-Enhanced Delivery

Most nanoparticles enter cells via endocytosis pathways [142]. The endocytosis-mediated internalization process includes fusion to early endosomes, maturation in late endosomes and degradation in lysosomes [143]. Therefore, escape from endosomes in the early stage is a key requirement for the efficient delivery of Cas9 into the nucleus. To induce the rapid release of nucleic acids or proteins into the cytoplasm, researchers have proposed several approaches for endosomal escape, including PEI-mediated osmotic pressure, liposome-mediated membrane fusion, pH-responsive polymer-mediated swelling, and cationic peptide-mediated membrane destabilization [144]. In this section, we focus on studies using chemicals and peptides for enhancing endocytosis or endosomal escape.

The peptide pardaxin, a membrane-penetrating peptide originally identified in the Red Sea sole [131], has been used for intracellular nonlysosomal trafficking of gene-editing components [145]. In this study, Jian and coworkers modified the surfaces of DOTAP-based cationic liposome with pardaxin. They demonstrated that pardaxin could facilitate uptake of liposome via a nonlysosomal pathway and transport them into the nucleus through the endoplasmic reticulum-nucleus pathway. FITC-labeled pardaxin-conjugated liposomes were found to localize to the endoplasmic reticulum rather than to lysosomes in MCF-7 cells. By circumventing the lysosomal route, this peptide modification protected plasmid DNA from degradation and prolonged the retention time of plasmids in cells. Although this study demonstrated the feasibility of avoiding lysosomal capture, further mechanistic studies assessing how pardaxin peptide drives localization of nanoparticles to the endoplasmic reticulum should be performed.

Glucuronylglucosyl-mediated endosomal escape has been studied for achieving efficient genome editing in the brain [132]. In this study, a dendrimer containing glucuronylglucosyl-β-CD conjugate was used as a Cas9/sgRNA ribonucleoprotein delivery system. A glucuronylglucosyl moiety, designed to act as a spacer, was inserted between the β-CD conjugate and dendrimer molecule, enhancing interaction of the dendrimer with the endosomal membrane, thereby destabilizing the endosomal membrane and enabling endosome escape. Intraventricular administration of Rosa26-targeted micelles loaded with Cas9/sgRNA ribonucleoprotein induced a gene editing efficiency of 5% at the injection site, a value ≈3-times higher than that for the Cas9/sgRNA complex alone.

Endosomal escape triggered by a cationic diethylenetriamine moiety has been studied for facilitating Cas9 mRNA and sgRNA delivery [133]. In this application, diethylenetriamine was conjugated to the side chain of amphiphilic polyaspartamide derivatives and complexed to Cas9 mRNA or sgRNA via electrostatic interactions. Diethylenetriamine moieties act as mRNA condensing agents and play a role in endosomal escape by destabilizing the endosomal membrane. Acidic pH-induced endosomal disruption may occur through a change in the protonation status of the endosome from monoprotonated at neutral pH (≈7.4) to deprotonated at acidic pH (≈5.5). Increasing the N/P ratio enhanced luminescence intensities in C2C12 and Neuro-2a cells transfected with luciferase-encoded mRNA/polyplexes, supporting a role for diethylenetriamine in the endosomal-escape effect. Luminescence signals of intracerebroventricularly administered luciferase-encoded mRNA/polyplexes in the mouse brain increased with increasing N/P ratio. However, additional mechanistic studies are needed to more clearly define the intracellular trafficking of Cas9 mRNA delivered via diethylenetriamine moiety-modified delivery systems. In addition, the genome-editing efficiency of this system should be demonstrated in animal models.

Endosomal disruption triggered by a helical cationic polypeptide has been studied for the delivery of plasmid DNA encoding Cas9 and sgRNA specific for PLK1 [126]. This cationic polypeptide, poly(γ-4-((2-(piperidin-1-yl)ethyl)aminomethyl)benzyl-L-glutamate) (PPABLG), is capable of binding and condensing plasmids without losing its helical structure, facilitating the efficient membrane-penetrating ability that underlies its role in promoting intracellular uptake and endosomal escape. Moreover, PPABLG was reported to retain its helical structure regardless of pH, ionic strength, and temperature, offering additional advantages for gene delivery. PEG-polythymine_40_ was further incorporated into PPABLG/plasmid DNA complexes through electrostatic interactions to enhance the stability of nanocomplexes. The Cas9-encoding plasmid, fluorescently labeled with YOYO-1 to facilitate monitoring, was found to be distributed throughout the cytoplasm of transfected HeLa cells, with low accumulation in endosomes, indicating the feasibility of using the helical peptide for endosomal disruption. Helical polypeptide-modified nanocomplexes (1 mg/kg plasmid), intratumorally injected into HeLa cell tumor-bearing mice, inhibited tumor growth compared with that in mice treated with phosphate-buffered saline. These nanocomplexes also downregulated expression of PLK1 and induced an indel frequency of 35% at the PLK1 genomic locus in the tumor tissue.

#### 4.1.2. Light-Enhanced Delivery

Among strategies for inducing endosomal escape or triggering the release of encapsulated cargoes is phototherapy. This approach, in which a stimulus is delivered by laser irradiation, has gained increasing attention owing to its unique spatial and temporal control properties, which allow drug activation at the desired place and time. A number of biomaterials have been developed for use in phototherapy-based delivery of small drugs or genes [146,147]. The light source used in phototherapy shows little or no harmful side effects on irradiated tissue compared with other stimuli such as radio radiation. Light-triggered release of gene-editing cargoes offers certain advantages. First, it enables remote control of gene editing performance at the target tissue. Second, it facilitates endosome escape or triggers degradation of the carrier to liberate the cargo.

Light-responsive drug delivery relies on two main activation mechanisms: photothermal and photodynamic. In photothermal activation, the irradiated material absorbs photons, which are converted to heat, resulting in a concurrent increase in temperature [148]. In contrast, photodynamic activation is based on the photochemical reaction of a light-absorptive reagent and ambient oxygen to generate reactive oxygen species (ROS) [149]. Both photothermal and photodynamic activation have been studied for intracellular delivery of Cas9/sgRNA plasmids.

Near-infrared (NIR) irradiation- and chlorin e6-triggered lysosome escape have been reported for the controlled release of Cas9 protein into the cytoplasm [134]. ROS generated by NIR irradiation in the presence of photosensitizers such as chlorin e6 are capable of destabilizing lysosome membranes, thereby releasing Cas9/sgRNA ribonucleoproteins from lysosomes. For this purpose, chlorin e6 was encapsulated into polymeric micelles, composed of a nitrilotriacetic acid–disulfanediyldipropionate–polyethyleneglycol–b-polycaprolactone copolymer, through hydrophobic interactions. His-tagged Cas9/sgRNA ribonucleoproteins targeting nuclear factor erythroid-2-related factor 2 (Nrf2) were bound to the nickel-terminated PEG block on micelles. In a CNE-2 xenograft mouse model intravenously injected with Nrf2-targeted micelles and exposed to NIR irradiation, the indel frequency for Nrf2 in tumor tissues was 31.2%, whereas mice treated with micelles lacking chlorin e6 showed no Nrf2 editing.

A photothermal release strategy has been studied for delivery of plasmid DNA encoding Cas9 and sgRNA specific for the PLK1 gene [127]. In this study, a Cas9/sgRNA plasmid was condensed with TAT peptide-conjugated gold nanoparticles, after which the complex core was coated with a lipid bilayer composed of DOTAP, DOPE, cholesterol, and DSPE-PEG2000. Owing to the photothermal feature of gold nanoparticles, the heat induced by irradiation with a 514-nm laser compromised the integrity of the lipid membrane and released 79.4% of payload plasmid DNA. Mice intratumorally injected with nanoparticles and then exposed to light showed significantly reduced tumor growth compared with nonirradiated mice injected with nanoparticles. Notably, the temperature at tumor tissues in this study was controlled at 42 °C or even less, ensuring that the tumor inhibition was attributable to PLK1 knockout rather than a photothermal effect. Irradiation of the tumor site with a 514-nm laser was found to trigger the release of Cas9 plasmids in a subcutaneous skin tumor model. From a translational perspective, the limited penetration of 514-nm light may pose a hurdle for therapy of deep tumor tissues. This strategy ultimately may be more suitable for gene editing in skin diseases, where the targeted tissue is accessible to this short wavelength.

A photolabile polymer has been exploited for intracellular delivery of plasmid DNA encoding Cas9 and sgRNA [135]. In this study, a semiconducting polymer derivative was grafted with PEI via thioester bond linkers. As thioester linkages are labile in the presence of ROS and the semiconducting polymer backbone produces ROS upon exposure to a 680-nm laser, laser irradiation of the thioester-linked semiconducting polymer-PEI is predicted to trigger ROS-mediated liberation of the linkage and release plasmid DNA. As expected, laser irradiation increased the gene editing efficiency of this polymer by almost 10-fold—from 0.9% to 8.7%. Upon local injection, this polymer/Cas9 polyplex showed a gene-editing efficiency in tumors that was 1.8-fold higher in the laser-irradiated group than in the nonirradiated group.

Fluorinated PEI modified with a photoresponsive semiconducting moiety has been reported as a delivery system for genome-editing components [37]. This derivative of fluorinated PEI with a semiconducting hydrophobic polymer and incorporated dexamethasone was designed to facilitate the release of genome-editing components into the cytoplasm by providing photoresponsive heat generation. Upon internalizing into the endosome, the polyplex reacts with laser light irradiation and escapes the endosome by inducing heat. The release of dexamethasone from the hydrophobic compartment of the polyplex assists nuclear-pore dilation and promotes nuclear entry of the Cas9/sgRNA plasmid, in this case, targeting the GFP gene. Due to the unique optical properties of the semiconducting polymer, this system can emit fluorescence at 950–1700 nm, allowing deep tissue imaging of nanoparticle distribution. The GFP gene-editing efficiency of this system, delivered via intratumoral injection, was demonstrated in mice bearing HCT116-GFP tumors.

#### 4.1.3. Glutathione-Triggered Delivery

Glutathione (GSH)-responsive delivery has been studied as a strategy for enhancing the release of genetic cargo and thus improving delivery and editing efficiency. The concentration of GSH in the cytosol (2–10 mM) is ≈1000-fold higher than that in the extracellular compartment (2–20 μM) [150,151]. One of the best-studied GSH-responsive linkers is the disulfide bridge, which can be a target for intracellular GSH-induced thiol-disulfide exchange [152].

A GSH-reducible synthetic lipid nanoparticle has been reported for the delivery of Cas9/sgRNA ribonucleoprotein complexes [128]. In this study, 8-O14B was inserted as a GSH-responsive component into lipid nanoparticles composed of DOPE, cholesterol, and C16-PEG2000-ceramide. 8-O14B has amine groups that provide positive charges; it also has disulfide bonds, and thus is degradable by GSH. Lipid nanoparticles containing 8-O14B were found to enter cells via clathrin-mediated endocytosis and escape endosomes. In EGFP-expressing HEK cells, the genome-editing efficiency of Cas9/sgRNA ribonucleoprotein complex delivered by 8-O14B-modified lipid nanoparticles was demonstrated by the loss of target gene expression.

Disulfide bond-based synthetic lipids have been studied for the delivery of Cas9 mRNA and sgRNA specific for the PCSK9 gene in a mouse model [136]. In this study, cationic lipid nanoparticles composed of cholesterol, DOPE, and DSPE-PEG-2000 were formulated to contain the synthetic lipid BAMEA-O16B, which has an ionizable amine head group and GSH-reducible disulfide linker that is presumed to be degraded by GSH in the cytosol. Although the presence of BAMEA-O16B did not affect the cellular delivery efficiency of lipid nanoparticles, it did affect the gene-editing efficiency of transfected systems. In a mouse model, the delivery of Cas9 mRNA/sgRNA delivery using BAMEA-O16B-containing lipid nanoparticle was shown to reduce serum PCSK9 protein levels compared with lipid particles without the GSH-reducible disulfide component.

GSH-responsive, disulfide bond-bearing cationic block copolymers have been studied for delivery of gene-editing Cas9/sgRNA ribonucleoproteins targeting GFP [137]. These polyplexes were constructed from poly(aspartic acid-(2-aminoethyl disulfide)-(4-imidazolecarboxylic acid))−PEG possessing imidazole residues and disulfide bonds, and negatively charged DNA, mRNA, and Cas9/sgRNA ribonucleoprotein were incorporated through electrostatic interactions. At a GSH concentration of 1 mM or higher, very few GFP-expressing target cells were detected.

In another application of polymeric nanoparticles designed to deliver Cas9/sgRNA, Cas9/sgRNA ribonucleoprotein complexes were coated with the GSH-degradable crosslinker N,N′-bis(acryloyl)cystamine, which was then polymerized by disulfide bond formation [112]. The resulting nanoparticles were found to degrade in the presence of GSH concentrations higher than 1 mM. Modification with the CPP, CYGRKKRRQRRR, further improved the gene-editing efficiency of the system.

The effectiveness of GSH-responsive systems depends on attention to detail in the design of GSH-responsive features. Since GSH is present in the blood and microenvironment of tumors, GSH-bonds can be partially broken during circulation in the bloodstream before the nanoparticles reach the high intracellular GSH conditions of the target tissue.

#### 4.1.4. pH-Responsive Delivery

Taking advantage of pH variations among tissues and intracellular organelles, researchers have intensively investigated pH-sensitive delivery systems. These studies have focused on the low (acidic) pH of tumor tissue and endosomes as triggers for activation of delivery systems [153,154]. Regardless of the cargo format—DNA plasmids, mRNA encoding Cas9/sgRNA or Cas9/sgRNA protein complexes—therapeutics designed to deliver CRISPR tools require cell membrane penetration, endosome escape, and/or release from carriers to execute their activities Once the delivery system enters the endolysosome, it may be degraded by lysosomal enzymes unless it escapes to the cytoplasm. To promote the release of genome-editing components from endolysosomes, researchers have developed various pH-responsive polymers.

One strategy for accomplishing pH-responsive delivery is fabrication of a polymer backbone that can be degraded under the acidic conditions of endolysosomes. In one recent study, orthoester linkages, which are readily broken under low pH conditions, were introduced into a fluorinated cationic polymer constructed from an ortho diamine ester monomer and branched crosslinker units complexed to plasmid DNA encoding Cas9/sgRNA [138]. Compared with standard PEI, the orthoester polymer mediated higher transfection efficiency in A549 lung cancer cells with lower cytotoxicity. The reduced toxicity of the orthoester polymer could be explained by the pH-sensitive degradation of polymer into subunit monomers, which prevents the accumulation of toxic cationic polymers in treated cells. The delivery of a pH-labile polymer complexed with plasmid DNA encoding Cas9 and a survivin-specific sgRNA was shown to sensitize the tumor to the anticancer drug, temozolomide, resulting in synergistic tumor suppression.

In another study, a pH-sensitive derivative of polylysine-g-poly(ethylene glycol) was developed for delivery of Cas9/sgRNA ribonucleoprotein targeting STAT3 [129]. Specifically, a 2,5-dihydro-2,5-dioxofuran-3-acetic acid linkage was inserted between poly-L-lysine and PEG to induce breakdown of the grafted polymer, PLys_100_-CAmPEG_77_, at the tumor microenvironment pH. Once the polyplex reaches the tumor tissue, it is proposed that the low pH in the tumor tissue triggers degradation of the linkage, stripping out the PEG shell and exposing the positive charge of the cationic particle for subsequent cellular internalization. After intravenous injection in mice, these polyplexes showed higher accumulation in tumor tissue and greater STAT3 knockout compared to treatment with pH-unresponsive polyplexes.

A series of pH-sensitive amino lipids containing modified amine head groups have been synthesized for delivery of Cas9 plasmid [139]. These multifunctional lipids are derivatives of (1-aminoethyl)iminobis[N-(oleoylcysteinyl-1-aminoethyl)propionamide] containing a protonatable amine head group, two cysteinyl units, and two lipid tails. These cationic lipids are able to condense DNA via amine groups and form stable particles through hydrophobic interaction and disulfide crosslinking. The hydrophilic head was also histidine-modified to alter the pKa of the lipid, thus facilitating pH responses of lipid nanoparticles. As the imidazole group of histidine has a pKa of approximately 6 [155], the histidine-modified lipid would be charged at lysosome pH owing to protonation of histidine. The resulting pH-sensitive lipoplex was shown to provide more efficient target gene knockout than Lipofectamine-based lipoplexes. Additional studies will be necessary to assess the knockout efficiency as well as toxicity of these amine lipids before application in humans.

### 4.2. Physical Delivery Strategies

In order to introduce therapeutic genome-editing components into cytoplasm, physical delivery strategies have been widely studied [156]. In receptor-mediated delivery strategies, most drugs are degraded and lose their functions in the lysosomes after endocytosis. However, physical delivery strategies are advantageous in that they can provide rapid and efficient delivery into the cytoplasm [157]. For this purpose, various stimuli including mechanical or electrical forces have been applied to transiently deform the cellular membrane [158]. The physical strategies have been actively applied to the intracellular delivery of genome-editing components.

Direct introduction of sgRNA/Cas9 complex into the cell though needle-based membrane pore formation was found to induce efficient genome editing. Nakamura and colleagues developed silicon-based nanoneedle arrays with nanoneedles of 200-nm diameter [140]. In this system, sgRNA and Cas9 protein complex was adsorbed on the hydrophobic surfaces of nanoneedles. Oscillation of the nanoneedle at an amplitude of 1.0 μm and a frequency of 5 kHz for 1 min was designed to release 0.1 pmol of the complex through fluidic shear force. In GFP-expressing HeLa cells, the nanoneedle-mediated delivery of GFP-targeting sgRNA and Cas9 complex reduced 32% of GFP expression. In FP10SC2 mouse breast cancer cells, nanoneedle-mediated delivery of type VI intermediate filament nestin-targeted sgRNA and Cas9 complex was shown to induce 15.4% of the target gene disruption.

The transient cell membrane deformation by microfluidic chip system also reported to induce genome editing [130]. Fabricated by photolithography and reactive ion etching, a microfluidic chip with nano-silicon-blade was developed. As the cells passed through the system, shear forces between cell membrane and the constriction on the nano-blade generated transient membrane holes, thereby allowing passive diffusion of sgRNA and Cas9 protein complex from surrounding matrix into cytoplasm. When GFP-expressing MDA-MB-231 cells were passing through the nano-blade system with GFP targeting sgRNA/Cas9, the percentage of GFP positive cells decreased to 18%. The system also provided robust gene editing in hematopoietic stem cells. The nano-blade system with p42 isoform of C/EBPα targeting sgRNA and Cas9 complex showed decrease of the target protein expression and induced myeloid proliferation behavior of the hematopoietic stem cells.

Beside nanoneedles, nanowires have been investigated for the delivery of genome-editing components [141]. In the study, gold nanowires with a diameter of 400 nm and a length of 2 μm generated propulsion under ultrasound field, facilitating penetration through the cell membrane. GFP-targeting sgRNA and Cas9 protein complex was immobilized on the nanowire through a reversible disulfide bond and the cargo was designed to be released by intracellular GSH. Under the application of ultrasound, sgRNA/Cas9-loaded nanowires were shown to reduce the expression of GFP in B16F10 cells.

Physical delivery strategies may have the unique advantage of inducing efficient genome editing in hard-to-transfect cells like hematopoietic stem cells. However, more studies are needed on the potential risk of altering cellular activity and viability. Physical puncture of cell membrane by the nanoneedle or ultrasound may result in the cell stress and damages. In addition, the applications of nanoneedle-based array or chip system have been limited for ex vivo use. In the future, the extension of physical strategies for in vivo applications needs to be studied.

## 5. Challenges and Future Perspectives

The discovery of the CRISPR/Cas9 genome-editing machinery has opened a new door for the treatment of various incurable diseases. In addition to fine-tuning genome-editing tools per se, it will be crucial to develop therapeutic delivery systems that are safe and efficient. Although a number of delivery systems, including lipid, polymer, peptide/protein, and natural-derived vesicles, have been studied for the delivery of CRISPR machinery, the field remains in its infancy. Additional maturation will be necessary to improve on limited gene-editing efficiency, nonspecific delivery, and unknown toxicities associated with novel materials.

One of the main challenges in clinical translation of genome-editing technology is off-target effects at both the genetic level, caused by CRISPR tools, and at the cellular level, due to inadequate delivery systems. New generations or variants of CRISPR tools have been, and continue to be, developed to address the issue of genetic level off-target effects (i.e., mutations). However, off-target side effects are predominantly driven by the specificity—or more specifically, the lack of specificity—of the delivery system. Most current studies investigate gene-editing efficiency at the in vitro level, where gene-editing effects can easily be achieved using homogeneous cells in culture. However, when applied in a living body, where targeted tissues are typically surrounded by normal tissue, nonspecific delivery is largely unavoidable. To reduce off-target effects, researchers should pay greater attention to the design of targeting systems for delivery of genome-editing components.

A number of strategies have been investigated for tailoring vectors to possess specific delivery features, including the use of pH-responsive or photosensitive nanomaterials. The general principle underlying these strategies is that delivery is triggered by local stimuli within the target tissue, whether intrinsic, such as tumor microenvironment pH, or extrinsic, such as focused light irradiation. Activation of genome-editing carriers under these conditions may result in gene editing of all cells in the tissue, including normal immune cells and stromal cells. Therefore, the greater the specific targeting to disease cells or cells of interest, the better the safety profile of the stimulus-responsive delivery system. Accordingly, a stimulus-responsive nanomaterial modified to contain a ligand specific for a certain cell type would promise fewer off-target effects. Choosing specific markers that are uniquely expressed in the cells of interest would be ideal. However, phenotypic heterogeneity among targeted cells likely makes single-ligand targeting problematic. Thus, it may be that rational combinations of two or more ligands would more effectively promote tier-specific delivery.

Regardless of targeting specificity, the nonspecific uptake of carriers by immune cells, as a part of their foreign substance-clearance mission, poses a challenge. Notably, nonspecific uptake of Cas9 nanoparticles may generate unexpected mutations in immune cells, possibly potentiating the lethal toxicity caused by deleterious off-target mutations; even cancer risks cannot be excluded. In this latter context, two independent studies have raised concerns of cancer risks associated with selection for a p53-deficiency in Cas9-edited cells. These studies revealed that p53 antagonizes Cas9 activity. Thus, cells that are successfully edited by Cas9 may be prone to mutagenesis and possible p53-antagonized signaling pathways that could result in tumor growth [159,160].

To overcome these challenges, future research efforts should address approaches for preventing nonspecific uptake by immune cells. PEG modification to mask the delivery system from protein absorption, as well as immune cell scavenging might be possible strategies for reducing uptake by immune cells [161]. Recent studies have reported that carriers decorated with CD47 protein, a natural ligand that provides a “do not eat me” signal, can evade phagocytosis by immune cells [162]. Strategies for modifying carriers with immune-escaping ligands can be extended to prevent possible off-target immunological side effects of genome-editing delivery systems in the future.

The route of administration of Cas9/sgRNA delivery systems in clinical practice needs to be considered carefully. Systemic administration may allow particles to be deposited in deep targeted tissue, but may also be the most likely to cause unexpected gene-editing side effects owing to its large volume of distribution compared with other administration routes. Numerous studies have evaluated genome-editing efficacy using intravenous injection. These studies have reported accumulation of carriers in targeted tissue and demonstrated correlations between targeted gene knockout and disease relief in various animal models. However, there is still little evidence that the genomes of other vital organs, such as liver, lung or kidney—where most nanocarriers are deposited—remain intact.

From the perspective of safety and accessibility, local administration routes, such as ocular delivery or intratumoral injection, are more amenable to clinical translation. Successful tumor suppression and gene knockout have been observed following intratumoral administration in various studies [127,135]. Along these lines, intravitreal injection of Cas9/PAMAM polymeric nanoparticles has been used to knock in therapeutic genes in the retina [38], and local infusion of Cas13a/crRNA/lipid-coated polymeric nanoparticle into the bladder cavity has been used to reduce systemic off-target effects. These strategies promise safe gene-editing delivery systems for specific types of disease, including skin cancer, head and neck cancer, and retinal degeneration diseases.

Beyond the justifiable emphasis on editing efficiency, developing reliable assays for detecting and screening the effects of editing post therapy—even tracking minor changes in the genetic status of vital organs—is also an important consideration. The development of gene-sequencing techniques is developing at an accelerating pace. Such analyses, specifically RNA sequencing, have been applied across tissues to generate a transcriptome atlas of normal tissues in mouse and rat [163,164]. These studies provide a solid background for the development of reliable methods for monitoring systemic off-target effects of genome editing.

Unlike other systems for delivering specific siRNAs, miRNAs, or plasmid DNA, delivery systems for genome-editing components employ diverse combinations of delivery materials. In some studies, plasmid DNA encoding Cas9 and sgRNA was used. In these cases, Cas9 expression and sgRNA complexation needs to occur in the cytoplasm, and the complex must enter the nucleus for gene editing. This single-plasmid DNA-delivery system offers the advantage of simplicity, but the complex kinetics of Cas9 expression, complexation with sgRNA, and nuclear localization may reduce the efficiency of genome editing.

In some studies, Cas9 protein was complexed with sgRNA, and the ribonucleoprotein was delivered by liposomes or polymeric systems. This system may be advantageous because it eliminates the need for a cytosolic Cas9 expression step, but achieving sufficient loading of Cas9 ribonucleoprotein into various delivery systems remains a challenge. Development of a method for increasing the loading efficiency of Cas9 ribonucleoprotein would be one future research direction. A lingering issue with this approach is that injection of the foreign Cas9 protein into the body can induce an immunogenic response.

The possibility of an immune response to CRISPR/Cas9 components is a general concern in patients receiving this therapy. As Cas9 protein originates from the immune system of bacteria, it is inherently immunogenic in humans. Consistent with this, several studies have reported detecting serum immunoglobulin against Cas9 in 60–80% of the studied population [165]. The material used for the Cas9 protein delivery system should have low immunogenicity and a limited adjuvant effect so as to prevent further immune responses to Cas9. Chitosan is known as a STING (stimulator of IFN genes) agonist that induces the maturation and activation of dendritic cells [166]. Thus, the use of chitosan as delivery system for Cas9 protein needs to be carefully assessed from the standpoint of immunogenicity. Several synthetic cationic lipids have been reported to have an agonist effect on Toll-like receptors [167,168]. Therefore, the possibility of undesirable immune stimulation should be screened in advance, particularly for delivery of Cas9 in protein form.

Additional strategies for minimizing possible immune responses to Cas9 protein should also be considered. Codelivery of dexamethasone, as investigated by Chen and colleagues may be a possible solution for reducing immune responses against the Cas9 protein [37]. Dexamethasone coencapsulated in this polymeric system acts as a nuclear pore-dilating agent to enhance the trans-localization of DNA plasmids into the nucleus. However, dexamethasone can also act as an immune suppressor and induce immune tolerance to Cas9 protein. This tolerance mechanism has been reported in previous studies investigating tolerogenic vaccine concepts [169,170].

Natural vectors derived from extracellular vesicles are an emerging platform for delivery of genome-editing tools. Although these natural vesicles are superior in terms of biocompatibility and the homotropism-based targeting, they pose several challenges that need to be addressed before translation to clinical practice. First, the isolation of exosomes from donor cells may vary from batch to batch owing to variability in cell type, growth phase, and cell cycle. Quality control efforts should be applied not only to the final product but also to the donor cell source. Second, the bioactivity of cell-derived vesicles is a reflection of the synergistic effects of all components that make up the vesicles. However, current studies still lack a reliable method for characterizing the entire molecular repertoire of natural vesicles. High-throughput methods for screening cellular membrane components, such as proteomic, lipidomic, or glycomic analyses, coupled with mass spectroscopy and advanced algorithm analyses [171,172,173], hold promise in this context. Finally, studies supporting regulatory perspectives need to be done for approval of genome-editing components for future medicine [174].

## 6. Conclusions

In this review, we summarized the status of nonviral systems for delivery of genome-editing components. Various materials and strategies have been exploited for promoting editing efficiency and minimizing off-target genome editing. With the emergence of novel materials developed in parallel with new generations of CRISPR tools, the door to complete treatment of incurable diseases is closer to opening than ever. With the recent announcement of the award of the prestigious Nobel Prize to researchers responsible for developing the CRISPR/Cas9 system as a genome-editing tool, the race for Cas9 applications is on. Once long-term safety issues and clinical-related problems of CRISPR delivery are addressed, the first products of these powerful tools could become available for disease treatment in humans. How long before the promise of this CRISPR/Cas9 system can be realized will ultimately depend on how rapidly CRISPR delivery-system development efforts progress in resolving these remaining issues.

## Figures and Tables

**Table 1:** Nanovesicle materials for CRISPR/Cas9 and sgRNA delivery.

Material	Composition	Cas9	sgRNA	Target Gene	Editing Type	Target Disease	Ref
Lipid	DOTAP, DOPE, DSPE-PEG, Chol	Plasmid DNA	Plasmid DNA	HPV16E6, E7	Knockout	Cervical cancer	[30]
	DOTAP, DOPE, DSPE-PEG	Plasmid DNA	Plasmid DNA	Iduronidase	Knock-in	Mucopolysaccharidosis type I	[31]
	cKK-E12, DOPE, Chol, C14-PEG	mRNA	sgRNA	PCSK9	Knockout	Hypercholesterolemia	[32]
	Protamine, chondroitin sulfate, DOTAP, DOPE, DSPE-PEG	Plasmid DNA	Plasmid DNA	PLK1	Knockout	Melanoma	[33]
	Gold nanoparticle, TAT, DOTAP, DOPE, DSPE-PEG	Protein	Plasmid DNA	PLK1	Knockout	Melanoma	[34]
	MSN, DOTAP, DOPE, DSPE-PEG, Chol	Protein	sgRNA	PCSK9, Apolipo-protein C3, Angiopoietin-like 3	Knockout	Hypercholesterolemia	[35]
Polymer	PEGylated hyaluronan polymer with R8-RGD tandem peptide, fluorinated polyethyleneimine	Plasmid DNA	Plasmid DNA	MTH1	Knockout	Ovarian cancer	[36]
	Semiconducting polymers composed of eicosane, PEG, diketopyrrolopyrrole and fluorinated PEI	Plasmid DNA	Plasmid DNA	MTH1	Knockout	-	[37]
	Branched β-CD-PEI/Ad-PAMAM/Ad-PEG-TAT	Plasmid DNA	Plasmid DNA	Retinoschisin 1, GFP	Knock-in	X-linked juvenile retinoschisis	[38]
	Quaternary ammonium-terminated poly(propylene oxide)/Pluronic F127	Plasmid DNA	Plasmid DNA	HPV18-E7	Knockout	-	[39]
Extracellular vesicle	Exosome from SKOV3	Protein	sgRNA	Poly (ADP-ribose) polymerase 1	Knockout	Ovarian cancer	[40]
	Exosome modified with TLS11a aptamer-Chol	Protein	sgRNA	WNT10B	Knockout	Hepatocellular cancer	[41]
	Microvesicle	Protein	sgRNA	IQGAP1	Knockout	Hepatocellular cancer	[42]
	Microvesicle with anti-CD19 CAR	Protein	sgRNA	MYC	Knockout	Lymphoma	[43]
Peptide/protein	R7L10 peptide-based micelle	Protein	sgRNA	β-secretase 1	Knockout	Alzheimer’s disease	[44]
	Poly-L-arginine	Protein	sgRNA	GFP, Nuclear Receptor Interacting Protein 1	Knockout	Obesity	[45]
	VLP composed of VSV-G	Protein	sgRNA	GFP	Knockout	-	[46]
	VLP composed of Gag-Cas9 fusion, VSV-G, BaEVRLess, sgRNA	Protein	sgRNA	EMX1, DDX3, Tyrosinase, HPD	Knockout	Hereditary tyrosinemia type I (HT1)	[47]
	VLP composed of VSV-G, FKBP12-Gag, FRB-Cas9	Protein	sgRNA	Dystrophin	Knockout	Duchenne muscular dystrophy	[48]

PCSK9, protease proprotein convertase subtilisin/kexin type 9; PLK1, polo-like kinase 1; MTH1, mutT homolog 1; GFP, green fluorescent protein; IQGAP1, IQ motif-containing GTPase-activating protein 1; EMX1, empty spiracles homeobox 1; DDX3, DEAD-box RNA helicase 3; HPD, hydroxyphenylpyruvate dioxygenase.

**Table 2:** Targeted delivery systems for genome editing.

Targeting	Material	Composition	Cas9 Type	sgRNA Type	Target Gene	Editing Type	Target Disease	Ref
Chemical ligand-based targeting	Lipid	Lipid shell composed of Gal-PEG-DSPE, DOTAP, DOPE, Chol, TAT with AuNP	Protein	sgRNA	PCSK9	Knockout	High LDL cholesterol	[111]
	Polymer	Polymeric nanoparticle composed of cationic, anionic, imidazole, biodegradable crosslinker, acrylate-mPEG-ATRA	Protein	sgRNA	Stop-tdTomato	Knockout	-	[112]
	Lipid	Liposome composed of DOTAP, cholesterol, folate-PEG-succinyl-Chol	Plasmid DNA	Plasmid DNA	DNMT1	Knockout	Ovarian cancer	[113]
Peptide-based targeting	Lipid	Liposome-templated hydrogel nanoparticle composed of palmitoyl-transportan-iRGD	Protein	sgRNA	GFP	Knockout	-	[114]
	Lipid	Liposome-templated hydrogel nanoparticle composed of PEI-CD, PEI-AD, mHph3, iRGD	Protein	sgRNA	PLK-1	Knockout	Brain cancer	[115]
	Polymer	Core-shell polymeric nanoparticle composed of PF33, hyaluronan, RGD-R8-PEG	Plasmid DNA	Plasmid DNA	MTH1	Knockout	Ovarian cancer	[36]
Antibody and aptamer ligand-based targeting	Protein	Protamine-based nanoparticle composed of CaCO3, As1411/TAT functionalized carboxymethyl chitosan	Plasmid DNA	Plasmid DNA	CTNNB1	Knockout	-	[116]
	Protein	Protamine-based nanoparticle composed of CaCO3, As1411/mucin 1 aptamer functionalized heparin	Plasmid DNA	Plasmid DNA	Focal adhesion kinase	Knockout	-	[117]
	Polymer	Peptide-lipid micelle composed of PEG-PEI-Chol, LC09 aptamer	Plasmid DNA	Plasmid DNA	VEGF-A	Knockout	Osteo-sarcoma	[118]

ATRA, all-trans retinoic acid; DNMT1, DNA-methyltransferase 1; GFP, green fluorescent protein; PLK-1, polo-like kinase 1; MTH1, mutT homolog 1; CTNNB1, β-catenin; VEGF-A, vascular endothelial growth factor A.

**Table 3:** Strategies for delivering CRISPR/Cas9 and sgRNA.

Strategy	Mechanism	Material	Composition	Cas9 Type	sgRNA Type	Target Gene	Editing Type	Ref
Endosomal escape	Cationic charge-induced membrane disruption	Liposome	Pardaxin peptide-modified cationic liposome	Plasmid DNA	Plasmid DNA	CDC6	Knockout	[131]
		Polymer	Glucuronylglucosyl-β-cyclodextrin conjugated dendrimer	Protein	sgRNA	Rosa26	Knockout	[132]
		Polymer	Cationic diethylenetriamine-conjugated amphiphilic polyaspartamide derivatives	mRNA	sgRNA	Luciferase	Knockout	[133]
		Polypeptide/polymer	α-helical polypeptide incorporating PEG-polythymine 40-based nanocomplex	Plasmid DNA	Plasmid DNA	PLK1	Knockout	[126]
Photo-responsive delivery	Photodynamic effect	Polymer	Chlorin e6-loaded/cationic iRGD-modified copolymer-coated polymeric micelle	Protein	sgRNA	Nrf2	Knockout	[134]
		Polymer	PEI-thioester-semiconducting polymer	Plasmid DNA	Plasmid DNA	GFP	Knockout	[135]
	Photothermal effect	Lipid	Lipid coated-gold NP-TAT peptide	Plasmid DNA	Plasmid DNA	PLK-1	Knockout	[127]
		Polymer	Semiconducting polymers composed of eicosane, PEG, diketopyrrolopyrrole, and fluorinated PEI	Plasmid DNA	Plasmid DNA	MTH1	Knockout	[37]
GSH-responsive delivery	GSH-mediated bond cleavage	Lipid	Cationic lipid nanoparticle composed of 8-O14B, DOPE, Chol, C16-PEG-ceramide	Protein	sgRNA	GFP	Knockout	[128]
		Lipid	Cationic lipid nanoparticle composed of BAMEA-O16B, DOPE, DSPE-PEG, Chol	mRNA	sgRNA	PCSK9	Knockout	[136]
		Polymer	Cationic block copolymer composed of poly(Asp-AED-ICA)-PEG	Protein	sgRNA	mCherry	Knockout	[137]
		Polymer	Polymeric nanoparticle composed of cationic, anionic, imidazole, biodegradable crosslinker, acrylate-mPEG-ATRA	Protein	sgRNA	Stop-tdTomato	Knockout	[112]
pH-responsive delivery	pH-dependent degradation	Polymer	Ortho ester polymer/PEI	Protein	sgRNA	Survivin	Knockout	[138]
		Polymer	PLys_100_-CAmPEG_77_	Protein	sgRNA	STAT3, RUNX1	Knockout	[129]
	pH-dependent charge reversion	Lipid	1-Aminoethyl)iminobis [N-(oleoylcysteinyl-1- aminoethyl)propionamide lipid	Plasmid DNA	Plasmid DNA	GFP	Knockout	[139]
Physical delivery	Mechanical membrane deformation	Nanoneedle	Silicon based atomic force microscope cantilever type nanoneedle	Protein	sgRNA	GFP, Nestin	Knockout	[140]
		Nanoblade	Photolithography and reactive ion etching fabricated microfluidic chip with silicon nanoblade	Protein	sgRNA	C/EBPα	Knockout	[130]
	Ultrasound mediated membrane penetration	Metal	Cysteine modified asymmetric gold nanowire	Protein	sgRNA	GFP	Knockout	[141]

CDC6, cell division control protein 6; PLK1, polo-like kinase 1; Nrf2, nuclear factor erythroid-2-related factor 2; VEGFR2, vascular endothelial growth factor receptor 2; GFP, green fluorescent protein; PCSK9, proprotein convertase subtilisin/kexin type 9; STAT3, signal transducer and activator of transcription 3; RUNX1, runt-related transcription factor 1; C/EBPα, CCAAT enhancer binding protein alpha.

## Abbreviations

**Abbreviation**	**Full Name**
ANGPTL3	Angiopoietin like 3
APOC3	Apolipoprotein C
ASGP	Asialoglycoprotein
Cas	Clustered regularly interspaced short palindromic repeats-associated protein
CDC6	Cell division control protein 6
C/EBPα	CCAAT enhancer binding protein alpha
Chol-PEG	Polyethylene glycol-linked cholesterol
CPP	Cell-penetrating peptide
CRISPR	Clustered regularly interspaced short palindromic repeats
crRNAs	CRISPR RNAs
CTNNB1	β-catenin
DDX3	DEAD-box RNA helicase 3
DNMT1	DNA-methyltransferase 1
DOPE	1,2-dioleoyl-sn-glycero-3-phosphoethanolamine
DOTAP	1,2-dioleoyl-3-trimethylammoniumpropane
DSPE-PEG-2000	1,2-distearoyl-sn-glycero-3-phosphoethanolamine-N-[amino(polyethyleneglycol)-2000]
EGFP	Enhanced green fluorescent protein
EMX1	Empty spiracles homeobox 1
FKBP	FK506-binding protein
FRB	FKBP-rapamycin binding domain
GFP	Green fluorescent protein
GPX4	Glutathione peroxidase-4
GSH	Glutathione
HPD	Hydroxyphenylpyruvate dioxygenase
HPV16 E6/E7	Human papillomavirus 16 E6/E7
ICAM1	Intercellular adhesion molecule 1
IDUA	α-L-iduronidase
IQGAP1	IQ motif-containing GTPase-activating protein 1
miRNA	MicroRNA
MTH1	MutT homolog 1
NIR	Near-infrared
Nrf2	Nuclear factor erythroid-2-related factor 2
PAMAM	Polyamidoamine
PARP1	Poly (ADP-ribose) polymerase-1
pCas9	Plasmid DNA encoding clustered regularly interspaced short palindromic Repeats-associated protein 9
PCSK9	Protease proprotein convertase subtilisin/kexin type 9
PD-L1	Programmed death-ligand 1
PEG	Polyethylene glycol
PEI	Polyethyleneimine
PLK1	Polo-like kinase 1
PPABLG	Poly(γ-4-((2-(piperidin-1-yl)ethyl)aminomethyl)benzyl-L-glutamate)
psgRNA	Plasmid DNA encoding single-guide RNA
RHBDF1	Rhomboid 5 homolog 1
ROS	Reactive oxygen species
RUNX1	Runt-related transcription factor 1
sgRNA	Single-guide RNA
siRNA	Small inhibitory RNA
STAT3	Signal transducer and activator of transcription 3
STING	Stimulator of IFN genes
TALENs	Transcription activator-like effector nucleases
tracrRNAs	Trans-activating CRISPR RNAs
TYR	Tyrosinase
VEGF-A	Vascular endothelial growth factor A
VEGFR2	Vascular endothelial growth factor receptor 2
VLP	Virus-like particle
VSV	Vesicular stomatitis virus
ZFN	Zinc-finger nuclease
β-CD	β-cyclodextrin
